# Prognostic Circulating Cytokine Panels for Metronomic Chemotherapy in Metastatic Gastrointestinal Cancer: Exploratory Pharmacodynamic Biomarker Analysis of the Phase II COMET Trial

**DOI:** 10.3390/cancers18111762

**Published:** 2026-05-28

**Authors:** Maria Laura Manca, Paola Orlandi, Giacomo Allegrini, Marta Banchi, Arianna Bandini, Robert A. Kirken, Guido Bocci

**Affiliations:** 1 Department of Clinical and Experimental Medicine, University of Pisa, 56126 Pisa, Italy; laura.manca@unipi.it (M.L.M.); paola.orlandi@unipi.it (P.O.); 2 Division of Medical Oncology, Leghorn Hospital, 57124 Leghorn, Italy; giacomo.allegrini@uslnordovest.toscana.it; 3 Department of Translational Research and New Technologies in Medicine and Surgery, University of Pisa, 56126 Pisa, Italy; marta.banchi@phd.unipi.it (M.B.); arianna.bandini@phd.unipi.it (A.B.); 4 Department of Biological Sciences, University of Texas at El Paso (UTEP), El Paso, TX 79968, USA; rkirken@utep.edu

**Keywords:** metronomic chemotherapy, gastrointestinal cancer, biomarkers, precision medicine, IL-16, MCP-4, THBS-2

## Abstract

Background/Objectives: Metronomic chemotherapy offers a well-tolerated option for heavily pretreated metastatic gastrointestinal cancer patients, but reliable prognostic biomarkers for patient selection are lacking. This study aimed to identify exploratory circulating cytokine signatures associated with outcomes in patients treated with metronomic chemotherapy. Methods: We analyzed plasma samples from 34 patients enrolled in the COMET trial (EudraCT 2007-000065-38), a phase II study of metronomic UFT, cyclophosphamide, and celecoxib. An 88-cytokine Luminex^®^ (Luminex Corporation, Austin, TX, USA) panel was measured at baseline and at four treatment timepoints. Partial least squares discriminant analysis identified candidate biomarkers, followed by systematic combinatorial analysis using Manciu’s method to construct 3-cytokine composite risk scores. Results: Twenty-one patients (61.8%) experienced progressive disease and 13 (38.2%) achieved stable disease. Six biomarkers showed significant discriminative power: IL-16, MCP-4, THBS-2, Eotaxin-1, PDGF-AB/BB, and TRAIL. Three 3-cytokine panels achieved statistically significant risk stratification (all *p* < 0.05), with hazard ratios for overall survival ranging from 2.59 to 6.24. For the representative IL-16 + MCP-4 + THBS-2 panel, high-risk patients showed a median PFS of 2.0 vs. 4.0 months (HR 3.24, *p* = 0.0046) and a median OS of 5.8 vs. 11.1 months (HR 4.19, *p* = 0.0010). Conclusions: This exploratory pharmacodynamic biomarker analysis identifies three 3-cytokine panels associated with prognostic risk stratification in metronomic chemotherapy for metastatic gastrointestinal cancer. As this single-arm trial cannot distinguish prognostic from predictive value, findings are hypothesis-generating. Prospective external validation is required before clinical translation, and exploration in combination with immune checkpoint inhibitors is warranted.

## Simple Summary

Metronomic chemotherapy—a regimen based on continuous low doses of anticancer drugs—represents a valuable treatment option for patients with advanced gastrointestinal cancers who have already received multiple lines of therapy. However, identifying in advance which patients are most likely to benefit from this approach remains an unsolved challenge. In this study, we analyzed blood samples from patients enrolled in the COMET clinical trial to identify protein markers—specifically cytokines, small signaling molecules measurable in plasma—associated with treatment outcomes at baseline, before therapy begins. Using a systematic computational strategy, we identified three panels of three cytokines each, all showing a statistically significant ability to stratify patients by prognostic risk. As this was a single-arm trial without a control group, these findings cannot establish whether the identified cytokines are truly predictive of the benefits from metronomic chemotherapy specifically, or whether they reflect the underlying differences in prognosis. These results are therefore hypothesis-generating and require prospective external validation before any clinical translation. They represent a step toward precision medicine in metronomic chemotherapy and provide a rationale for future biomarker-stratified trials.

## 1. Introduction

Metastatic gastrointestinal cancer remains a leading cause of cancer-related mortality worldwide [1]. In particular, colorectal cancer is usually treated with a combination of surgery, chemotherapy, targeted therapy, and, in selected patients, immunotherapy [2]. In the metastatic disease setting, combination chemotherapy (FOLFOX, FOLFIRI, CAPOX) plus biologics (bevacizumab, cetuximab, panitumumab, other VEGF/EGFR-targeted agents) is a standard [3], whereas a small subgroup with mismatch-repair deficiency/microsatellite instability responds very well to immune checkpoint inhibitors [4]. These treatments improve survival but frequently cause adverse drug reactions (ADRs) that affect quality of life and may limit therapy [5]. Moreover, conventional cytotoxic regimens provide limited benefit in heavily pretreated patients [6]. Although recent trials support precision oncology in gastrointestinal cancers, highlighting the durable benefits of some targeted and immune therapies in combination with chemotherapeutic drugs, the efficacy and the adverse drug reactions of these approaches are underscoring the need for biomarker-driven patient selection [2].

Metronomic chemotherapy, defined as the frequent administration of cytotoxic drugs at low but effective doses, has emerged as an attractive therapeutic option due to its favorable toxicity profile and potential for long-term disease stabilization in various settings of patients, including the elderly and pediatric patients [7,8]. The biological rationale for metronomic therapy extends beyond conventional cytotoxicity to include anti-angiogenic effects, immune system activation, and the disruption of the tumor microenvironment [9,10,11].

In gastrointestinal cancers, clinical trials have shown heterogeneous responses to metronomic chemotherapies. A recent meta-analysis highlights metronomic capecitabine as a potential treatment for hepatocellular carcinoma (HCC) in the advanced stage [12], while a systematic review of randomized trials by Chen et al. indicates that metronomic chemotherapy for metastatic colorectal cancer (mCRC) could improve progression-free survival in some patients and is relatively safe, but the advantages in overall survival are not clear [13]; therefore, this variability underlines the critical and urgent need for predictive biomarkers to optimize patient selection and avoid ineffective metronomic treatments, as well as, from the perspective of their potential use as a combination partner with immune checkpoint inhibitors (ICIs) for the treatment of patients with mCRC tumors, most of which are immunologically cold [14].

Traditional clinicopathological parameters, including performance status, tumor location, and previous treatments, provide insufficient discrimination for treatment selection. The advent of high-throughput molecular profiling technologies offers unprecedented opportunities to identify circulating biomarkers that reflect the complex interplay between host immunity, angiogenesis, and tumor biology and are relevant to metronomic therapy mechanisms [15,16].

In gastrointestinal cancer patients treated with metronomic therapies, several individual biomarkers have been investigated, including vascular endothelial growth factor (VEGF), thrombospondin-1 (TSP-1), and soluble vascular endothelial-cadherin (sVE-C) plasma levels, or CD133 gene expression in peripheral blood mononuclear cells [17,18], or the baseline plasmatic levels of Angiopoietin-2 and Chemokine (C-X-C motif) ligand 14 (CXCL14), as well as the induction of programmed cell death protein 1 (PD-1) [19], or plasma interferon gamma (IFNγ) and soluble CD40L [15], but systematic approaches to identify optimal biomarker combinations are still lacking. Indeed, no validated predictive biomarker panel exists for metronomic chemotherapy in GI cancer, especially regarding the host immunity involvement in the mechanism of action behind metronomic chemotherapy [20]. The identification of potential immune biomarkers of benefit could completely change the therapeutic approach, currently based on low-dose chemotherapy, especially if combined with novel immunotherapies. Based on this consideration, the present study is the first systematic combinatorial analysis of a high number of cytokines across different timepoints in this setting, analyzing new experimental data on plasma circulating proteins from the COMET trial, a phase II study investigating metronomic chemotherapy in gastrointestinal cancer [18]. In fact our study addresses these issues through a comprehensive approach encompassing: (i) the systematic univariate screening of 88 circulating cytokines; (ii) combinatorial analysis using Manciu’s method [16] to identify optimal biomarker panels; (iii) multivariate modeling with rigorous validation; and (iv) longitudinal biomarker dynamics analysis. Our primary hypothesis was that baseline cytokine profiles contain predictive information sufficient to stratify patients by survival outcome before treatment begins. We show that three 3-biomarker combinations achieve robust, statistically significant risk stratification—with the IL-16 + MCP-4 + THBS-2 panel as a representative example—suggesting that a simple plasma test performed at diagnosis could support more informed therapeutic decisions in this challenging patient population.

## 2. Materials and Methods

### 2.1. Study Design and Population

New experimental data on plasma circulating proteins were analyzed from the COMET trial [18]. The study was registered in the European Clinical Trial Database EudraCT (https://eudract.ema.europa.eu/ (accessed on 15 April 2026); EudraCT registration number 2007-000065-38). All patients provided written informed consent, and the study was conducted in accordance with the Declaration of Helsinki. Patients received oral UFT (tegafur-uracil) 100 mg twice daily continuously, plus cyclophosphamide (500 mg/m^2^ i.v. bolus on Day 1, then 50 mg daily p.o. continuously), plus celecoxib 200 mg twice daily, until disease progression or unacceptable toxicity. Response assessment was performed every eight weeks using RECIST 1.1 criteria.

### 2.2. Blood Samples

Peripheral blood samples were collected at baseline and at Days 28, 56, 84, and 112 of treatment in plasma separator tubes, processed within 10 min, and stored at −80 °C until analysis. Samples were available from all 38 enrolled patients; 34 (89.5%) met pre-specified quality criteria and were included in the biomarker analysis. Plasma was analyzed for 88 circulating cytokines (Table 1) using a Luminex^®^ platform with MILLIPLEX^®^ Configurable Human Cytokine/Chemokine/Growth Factor Panel kits (Merck KGaA, Darmstadt, Germany), according to the manufacturer’s instructions. QC Luminex protocol details: plasma volume, processing time, inter-assay reproducibility, calibration standards, normalization, and batch effects have been added in Supplementary Materials.

All primary biomarker analyses were performed on baseline (Day 0) plasma cytokine measurements only. Longitudinal data (Days 28, 56, 84, and 112) were analyzed separately and descriptively and were not used in the construction or validation of the prognostic panels.”

### 2.3. Statistical Analysis

Partial least squares discriminant analysis (PLS-DA) was applied to identify the most relevant biomarker subset associated with both progression-free survival (PFS) and overall survival (OS), using the variable importance in projection (VIP) index [21]. Optimal cutpoints for each candidate biomarker were then determined using a custom R implementation of the Contal and O’Quigley method [22], with multiple testing corrections. Kaplan–Meier survival analysis and Cox proportional hazards models were used to assess associations with PFS and OS [23,24]. The systematic evaluation of all possible combinations among the selected biomarkers was performed using Manciu’s combinatorial method with composite risk scoring [16]. Model performance was assessed using Harrell’s C-index [25] and the Akaike Information Criterion (AIC) [26]. Internal validation was performed by leave-one-out cross-validation (LOOCV) and bootstrap resampling (2000 replicates) for confidence interval estimation [27].

**Figure 1:**
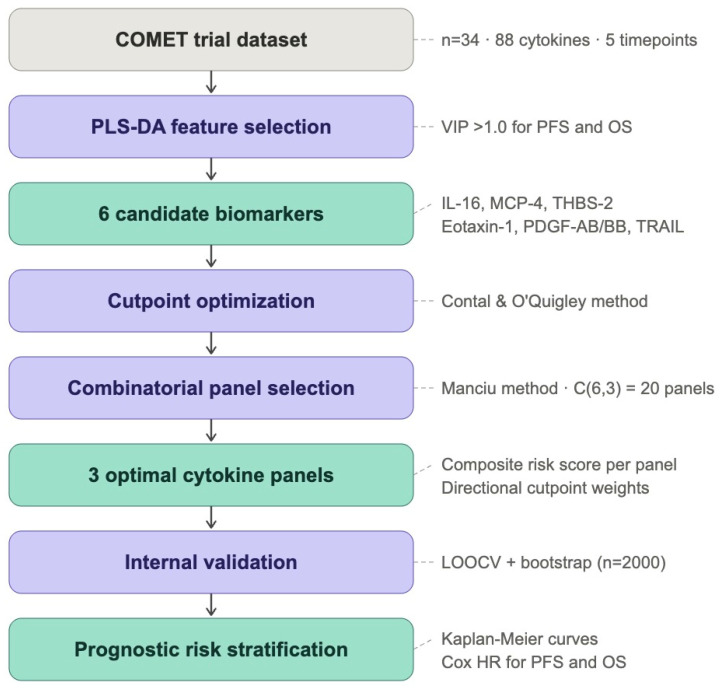
Schematic overview of the analytical pipeline. Starting from the full COMET dataset (n = 34, 88 cytokines, 5 timepoints), PLS-DA feature selection (VIP > 1.0) identified 6 candidate biomarkers, followed by Contal and O’Quigley cutpoint optimization, systematic combinatorial panel selection using Manciu’s method (C(6,3) = 20 panels evaluated), internal validation by LOOCV and bootstrap resampling (n = 2000), and final prognostic risk stratification by Kaplan–Meier curves and Cox proportional hazards regression.

It should be noted that feature selection (PLS-DA), cutpoint determination, and combinatorial panel construction were all performed within the same dataset; internal resampling therefore addresses variability in the final stratification step but does not fully correct for optimism introduced by the upstream selection pipeline. Results should accordingly be interpreted as hypothesis-generating within this cohort. A schematic overview of the full analytical pipeline is provided in Figure 1.

All primary analyses were performed using R version 4.5 [28] with the packages survival, survminer, mixOmics, and pROC. The custom R code implementing the Contal and O’Quigley cutpoint algorithm and Manciu’s combinatorial method is not publicly deposited but is available from the corresponding author upon reasonable request.

### 2.4. AI-Assisted Analysis

Analytical and manuscript preparation activities were performed with assistance from Claude Sonnet 4.5 (Anthropic, San Francisco, CA, USA), a large language model. AI assistance was used for the following specific tasks: (i) implementation and iterative refinement of the combinatorial biomarker scoring algorithm based on Manciu’s method; (ii) computation of composite risk scores and Harrell’s C-index for the three final cytokine panels; (iii) data visualization, including the generation of summary statistics and longitudinal trajectory tables; and (iv) drafting and structural editing of the manuscript text. AI assistance was not used for primary statistical analyses; PLS-DA, Contal and O’Quigley cutpoint optimization, Kaplan–Meier estimation, and Cox proportional hazards regression were all performed independently using R version 4.5 with packages survival, survminer, mixOmics, and pROC. All AI-generated outputs were critically reviewed, independently verified against R-derived results, and refined by the research team prior to inclusion. The AI system did not have access to individual patient-level data and did not make autonomous clinical or statistical interpretations. Human expertise and oversight remained central to all aspects of study design, data analysis, interpretation, and clinical conclusions.

## 3. Results

### 3.1. Patient Characteristics and Outcomes

A total of 34 patients with metastatic gastrointestinal cancers were included in the final biomarker analysis. The cohort size reflects the constraints inherent to the COMET trial: A single-center phase II study enrolling heavily pretreated patients with advanced gastrointestinal cancers between 2007 and 2009; 34 of 38 enrolled patients (89.5%) met pre-specified Luminex quality criteria and were included. While this sample size is acknowledged as a limitation (see Section 4), it is comparable to or larger than prior biomarker studies in this heavily pretreated population [15,17,18,19] and was sufficient to identify statistically significant risk stratification across three independent cytokine panels. The cohort comprised heavily pretreated individuals who had received at least two prior lines of therapy. Primary tumor types included colorectal cancer (n = 30, 88.2%), biliary tract cancer (n = 2, 5.9%), hepatocellular carcinoma (n = 1, 2.9%), and pancreatic cancer (n = 1, 2.9%). The predominance of colorectal cancer (88.2%) reflects the epidemiology of advanced gastrointestinal malignancies in this treatment-refractory setting; exploratory subgroup analysis restricted to colorectal cancer patients (n = 30) yielded results consistent with the full-cohort findings (Supplementary Table S6), and tumor type (CRC vs. non-CRC) was not a significant prognostic factor in either univariate (HR = 1.30, *p* = 0.634) or multivariate (HR = 1.66, *p* = 0.367) Cox regression, supporting the retention of the mixed-histology cohort for primary analysis. Two response categories were observed: 21 patients (61.8%) experienced progressive disease (PD) and 13 (38.2%) achieved stable disease (SD). No partial or complete responses were recorded in this advanced population.

### 3.2. Biomarker Selection

PLS-DA identified six circulating biomarkers with VIP scores >1.0 for both PFS and OS: IL-16, MCP-4, THBS-2, EOTAXIN-1, PDGF-AB/BB, and TRAIL. These biomarkers span distinct biological processes: IL-16 reflects pro-inflammatory immune activation [29]; MCP-4 and EOTAXIN-1 mediate the chemotaxis of immune effector cells [30,31]; THBS-2 and PDGF-AB/BB are involved in extracellular matrix remodeling and angiogenesis regulation [32,33]; and TRAIL regulates apoptotic signaling. Baseline median plasma concentrations were: IL-16 95.07 pg/mL (IQR 91.54–102.78), MCP-4 56.08 pg/mL (IQR 44.66–92.63), THBS-2 181.85 pg/mL (IQR 83.30–270.85), EOTAXIN-1 422.14 pg/mL (IQR 274.90–598.16), PDGF-AB/BB 108.76 pg/mL (IQR 47.73–266.30), and TRAIL 44.92 pg/mL (IQR 34.06–63.94). Comprehensive baseline values for all 88 cytokines are reported in Table 1 and Supplementary Table S5; longitudinal measurements at Days 28, 56, 84, and 112 are provided in Supplementary Tables S1–S4.

### 3.3. Longitudinal Biomarker Dynamics

Longitudinal analysis revealed distinct biomarker trajectories between PD and SD patients across the treatment period (Table 2a,b). MCP-4 showed progressive group divergence: PD patients exhibited sustained increases (+10% at Day 56, +54% at Day 112 relative to baseline), whereas SD patients remained stable (0% at Day 56, −7% at Day 112). THBS-2 increased substantially in both groups by Day 112 (>100% above baseline), but concentrations in PD patients were approximately four-fold higher than in SD patients throughout follow-up. EOTAXIN-1 showed a modest rise in PD patients, while SD patients maintained concentrations approximately twice as high as those observed in the PD group. PDGF-AB/BB levels declined progressively in SD patients during treatment, in contrast to PD patients. The interpretation of Day 84 and Day 112 timepoints is limited by reduced PD sample availability due to disease progression (n = 6 and n = 3, respectively).

### 3.4. Optimal Biomarker Combinations

Systematic combinatorial analysis using Manciu’s method identified three 3-biomarker combinations with statistically significant and clinically meaningful risk stratification for both PFS and OS (Table 3). For all biomarkers, values below the individual cutpoint—determined by the Contal and O’Quigley method—were associated with worse outcomes, with the single exception of THBS-2, for which values above the cutpoint identified the high-risk group. Composite risk scores incorporated directional weights accordingly.

**Figure 2:**
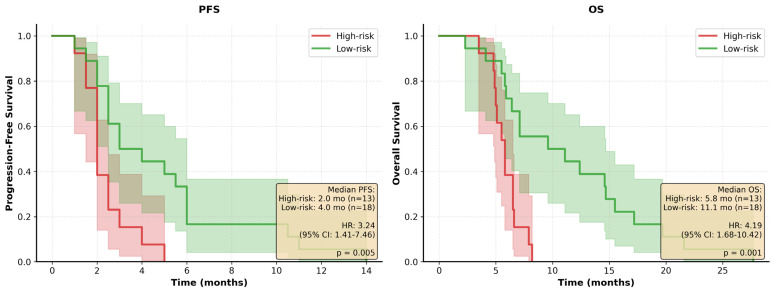
Kaplan–Meier survival curves for the IL-16 + MCP-4 + THBS-2 biomarker combination. Patients were stratified into high-risk (n = 13, red) and low-risk (n = 18, green) groups based on a composite risk score derived from baseline plasma concentrations. Shaded areas represent 95% confidence intervals.

**Figure 3:**
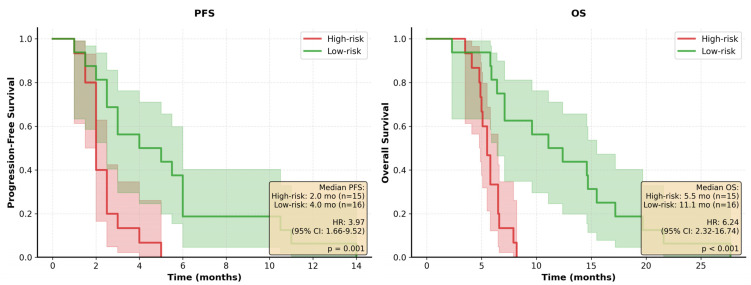
Kaplan–Meier survival curves for the MCP-4 + PDGF-AB/BB + THBS-2 biomarker combination. Patients were stratified into high-risk (n = 15, red) and low-risk (n = 16, green) groups. This combination achieved the highest hazard ratio for overall survival among the three panels. Shaded areas represent 95% confidence intervals.

**Figure 4:**
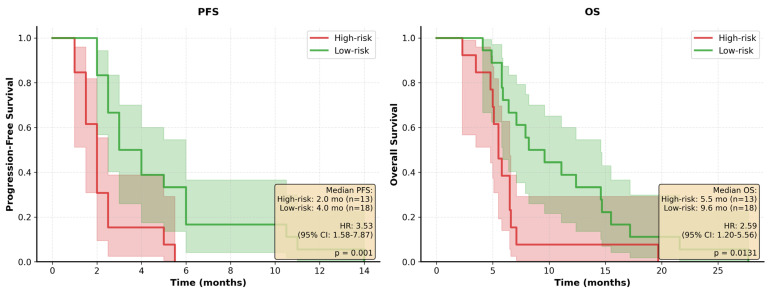
Kaplan–Meier survival curves for the EOTAXIN-1 + IL-16 + THBS-2 biomarker combination. Patients were stratified into high-risk (n = 13, red) and low-risk (n = 18, green) groups. Shaded areas represent 95% confidence intervals.

Internal validation by LOOCV and bootstrap resampling (2000 replicates) confirmed the robustness of risk stratification across all three combinations. The comparable discriminative performance of the three panels—with HR for OS ranging from 2.59 to 6.24 and all *p*-values < 0.05—indicates that THBS-2, together with at least one immune-related cytokine (IL-16, MCP-4, or EOTAXIN-1), constitutes a consistent predictive core in this patient population (Figure 2, Figure 3 and Figure 4). Univariate and multivariate Cox regression analyses adjusted for available clinical covariates (ECOG performance status and primary tumor type) are presented in Table 4; ECOG PS ≥ 1 vs. 0 was a significant independent prognostic factor for PFS (univariate HR = 3.37, 95% CI 1.13–10.02, *p* = 0.029; multivariate HR = 3.65, 95% CI 1.21–10.98, *p* = 0.021), while tumor type (CRC vs. non-CRC) was not significant (*p* = 0.634) (Supplementary Figure S1).

## 4. Discussion

Gastrointestinal cancers, particularly colorectal cancer, remain among the most common malignancies and leading causes of cancer death worldwide [34,35]. Approximately 22% of colorectal cancers present distant metastases at diagnosis, with 5-year survival rates of 14% [35]. For previously treated or elderly, frail patients with metastatic disease, approaches that provide symptomatic palliation while reducing tumor burden with minimal toxicity are crucial for maintaining quality of life [8,36]. This study represents the first comprehensive pharmacodynamic biomarker analysis of metronomic chemotherapy in gastrointestinal cancers, identifying predictive signatures that could facilitate patient selection by clinical oncologists for this therapeutic approach.

This is also the first systematic combinatorial biomarker analysis applied to metronomic chemotherapy in metastatic gastrointestinal cancer. To our knowledge, prior work on this patient population—including the earlier COMET-derived analysis by Valenzuela et al. [15], which examined individual cytokines in 31 patients at two timepoints—has been limited to single-marker approaches. The present analysis extends that work by screening 88 cytokines across five timepoints in 34 patients and applying Manciu’s combinatorial method [16] to systematically identify optimal multi-marker signatures. The consistency of discriminative performance across the three identified combinations—hazard ratios for OS ranging from 2.59 to 6.24, all with *p* < 0.05—reinforces the biological relevance of the identified markers rather than suggesting overfitting to a single solution.

Among the numerous plasma factors considered in our analysis, it certainly stands out that at least two factors linked to patient survival and response, namely EOTAXIN-1 and MCP-4, are involved in the chemo-attractive processes towards immune system cells. Indeed, it has been demonstrated that experimental mucosal-associated invariant T cell therapy in mice increases tumor EOTAXIN-1 and eosinophil-linked killing, illustrating that the CCL11–eosinophil axis can also be co-opted for antitumor immunity [37]. This would suggest a potential relationship between the ability to attract eosinophils and the greater efficacy of metronomic therapy’s antitumor immunity, given that patients with SD have double levels of plasma concentrations compared to the other group. Furthermore, MCP-4, which is known to support tumor progression by recruiting tumor-associated macrophages (TAMs) [38], was found stable in SD patients responding to metronomic chemotherapy. The recent study by Spehner et al. [19], investigating biological determinants of metronomic chemotherapy efficacy in chemo-refractory gastrointestinal cancers, similarly highlights the central role of immune microenvironment modulation, further supporting the plausibility of immune-related cytokines as predictive markers in this setting.

Since PDGF-BB drives angiogenesis by recruiting pericytes and vascular smooth muscle cells, sometimes inversely related to VEGF in experimental models [39], it is interesting to note that its levels in patients treated with metronomic chemotherapy and responding to therapy decrease over time, underlining the important role of angiogenic inhibition in metronomic treatment. Thrombospondin-2 (THBS-2) is an extracellular matrix glycoprotein increasingly recognized as a key driver of colorectal cancer (CRC) progression rather than a simple antiangiogenic factor [32,40]. Consistent with the original observation that thrombospondin-1 mediates the anti-angiogenic effects of low-dose metronomic chemotherapy [41], THBS-2 in our cohort appears to reflect a related but distinct pro-tumoral matrix remodeling program that metronomic therapy fails to suppress in non-responding patients. Indeed, higher basal levels were found in patients not responding to metronomic chemotherapy with a worse survival rate. This directional asymmetry—values above the THBS-2 cutpoint identifying the high-risk group, opposite to all other markers—has direct methodological implications: composite risk scores must incorporate biomarker-specific directionality, and THBS-2 cannot be treated interchangeably with immunological markers in the panel.

Colorectal cancer behavior, spread, and treatment response are strongly shaped by the tumor microenvironment (TME), the mix of immune cells, fibroblasts, blood vessels, extracellular matrix components around the tumor [42]. The colorectal cancer TME is a dynamic ecosystem that can either restrain or fuel tumor growth. Its immune cells, fibroblasts, vasculature, and extracellular matrix jointly determine progression, metastasis patterns, and response to modern therapies, particularly immunotherapy [43]. Directly targeting these TME components with metronomic chemotherapy could be a central strategy for more precise and effective treatment [44,45]. For this reason, the identified cytokine panels capture simultaneous information from complementary but biologically distinct axes—immune activation (IL-16), chemotaxis of effector immune cells (MCP-4, Eotaxin-1 via the CCL13–CCL11 chemokine axis), angiogenesis regulation (PDGF-AB/BB), and extracellular matrix remodeling (THBS-2)—on which metronomic chemotherapy efficacy could depend on. The composite risk score integrates signals from these interacting pathways, providing a more comprehensive readout of the tumor microenvironment than any single marker can offer and, above all, may suggest a rational approach for metronomic chemotherapy as an immunomodulatory partner for novel immunotherapies. Although the tumor-type-specific cytokine profiles remain an open question in our pilot study, the analyses seem to suggest that the host-immunity cytokine characterization found in CRC can be applied also to the GI full-cohort findings.

These results are particularly remarkable considering our cohort consisted entirely of heavily pretreated patients with metastatic disease who had failed at least two prior treatment lines [18]. In this challenging population, achieving response rates above 40% (SD) with well-tolerated oral regimens represents a clinically meaningful outcome, especially when combined with the ability to predict treatment benefit prospectively.

Metronomic chemotherapy, defined as the frequent administration of chemotherapeutic drugs at doses below the maximum tolerated dose, has emerged as an attractive therapeutic strategy in gastrointestinal cancers due to its favorable toxicity profile and potential for long-term disease stabilization [3,46]. Easy access, remarkable tolerability, and cost-effectiveness, along with notable activity in resistant tumors, have rendered metronomic therapy an intriguing area of oncology research, particularly for gastrointestinal malignancies but still lacking drug activity biomarkers for the personalization of the schedules [3,4]. Our biomarker panel offers a practical approach to precision medicine in the metronomic setting. From a technical standpoint, the Luminex^®^ multiplex immunoassay platform used in this study requires only 25 μL of plasma per patient, with a processing time of approximately 24 h including overnight incubation, and is already available in specialized oncology laboratory settings. As detailed in Supplementary Table S7, intra-assay and inter-assay precision for the six key biomarkers were within acceptable ranges (inter-assay %CV <20% for all analytes), supporting the technical reproducibility of the assay, and the accuracy with spike recovery is reported in Supplementary Table S8. The combination of three readily measurable plasma cytokines could therefore be implemented in routine clinical practice using existing laboratory infrastructure, without requiring novel or specialized platforms. The 8-week window for response assessment in current practice could be substantially shortened, potentially saving patients from ineffective treatments and associated healthcare costs [46]. Formal health-economic analyses and regulatory validation are beyond the scope of this hypothesis-generating study and will require prospective validation data; the present findings provide the biological and analytical rationale for the design of a prospective biomarker-stratified trial.

These results should be interpreted in the context of several limitations. First, the biomarker analysis was conducted retrospectively on a single-arm phase II trial with 34 evaluable patients, which constrains both statistical power and generalizability. Second, the single-center design limits the assessment of pre-analytical variability across institutions. Third, for IL-16 and EOTAXIN-1, the Contal and O’Quigley algorithm identified optimal cutpoints coinciding with the cohort median; while this is an expected outcome of the method in small datasets with approximately balanced outcome distributions, it underscores the importance of external validation with pre-specified cutpoints before any clinical translation. Fourth, internal validation by LOOCV and bootstrap, while appropriate for the sample size, cannot substitute for prospective validation in independent cohorts. Fifth, the study predates the current era of combined metronomic–ICI strategies; whether these biomarker panels retain predictive value in combination regimens remains an open question.

Sixth, the multi-step analytical pipeline—encompassing PLS-DA-based feature selection, Contal and O’Quigley cutpoint optimization, and combinatorial panel construction—was applied entirely within the same 34-patient dataset. Although internal resampling (LOOCV, bootstrap) was used, it addresses variability at the stratification step only and does not correct for optimism accumulated through upstream data-driven selections. Stronger safeguards such as nested cross-validation or permutation testing would be warranted in a future study with a larger sample.

Seventh, the longitudinal biomarker analysis presented here is descriptive in nature. More robust approaches—such as time-dependent Cox regression or linear mixed-effects models—would provide stronger evidence for dynamic biomarker trajectories associated with outcome. However, the markedly reduced sample size at later timepoints (Day 84: n = 6 for PD; Day 112: n = 3 for PD) precludes the reliable application of these methods in the present dataset, and longitudinal modeling is deferred to future studies with adequate follow-up samples.

Future research should prioritize prospective external validation in independent gastrointestinal cancer cohorts, ideally with pre-specified cutpoints derived from the present analysis. Exploration of these panels in the context of ICI combination strategies would be particularly timely, given the emerging rationale for metronomic chemotherapy as an immunomodulatory partner for checkpoint blockades in immunologically cold tumors such as microsatellite-stable mCRC [14]. Mechanistic studies clarifying the causal relationships between these cytokine profiles and metronomic therapy responses—particularly the contrasting roles of THBS-2 and the immune chemokines—would also strengthen the biological foundation for clinical implementation.

## 5. Conclusions

This comprehensive pharmacodynamic biomarker analysis of the COMET trial identifies three validated 3-cytokine panels—IL-16 + MCP-4 + THBS-2, MCP-4 + PDGF-AB/BB + THBS-2, and EOTAXIN-1 + IL-16 + THBS-2—capable of robust baseline risk stratification in metastatic gastrointestinal cancer patients treated with metronomic chemotherapy. The consistent discriminative performance across all three combinations, spanning immune activation, chemotaxis, angiogenesis, and extracellular matrix remodeling, reflects the multi-mechanistic pharmacodynamic profile of metronomic therapy and provides biological plausibility to the identified signatures.

These findings represent a step toward precision medicine in the metronomic setting: a simple multiplex plasma test at diagnosis could prospectively identify patients unlikely to benefit, avoiding ineffective treatment in a vulnerable, heavily pretreated population. External prospective validation in independent cohorts is required before clinical implementation, and an exploration of these signatures in the context of combination strategies with immune checkpoint inhibitors is warranted as a priority for future investigation.

## Figures and Tables

**Table 1:** List of the analyzed circulating plasma proteins, acronyms, mean ± standard deviation (SD), and median plasma concentrations (pg/mL) at Day 0 (baseline). IQR: interquartile range (25th–75th percentile). n = 34.

Analyzed Circulating Protein	Acronym	Other Acronyms	Mean (pg/mL) ± SD	Median (IQR)
Epidermal growth factor	EGF		113.90 ± 264.46	41.55 (29.40–73.40)
Fibroblast growth factor-2	FGF-2	bFGF	63.45 ± 8.85	60.80 (59.40–65.30)
Eotaxin-1	Eotaxin-1	CCL-11	583.02 ± 701.81	422.14 (274.90–598.16)
Transforming growth factor alpha	TGFα		9.86 ± 9.44	7.60 (3.10–14.22)
Granulocyte colony-stimulating factor	G-CSF	CSF-3	28.97 ± 23.48	23.23 (18.53–27.94)
Fms-like tyrosine kinase 3 ligand	FLT-3L	FLT3LG	25.98 ± 37.27	8.27 (1.89–39.64)
Granulocyte-Macrophage Colony-Stimulating Factor	GM-CSF	CSF-2	18.55 ± 14.01	15.07 (10.35–19.79)
Fractalkine	Fractalkine	CX3CL1	245.16 ± 994.82	57.65 (39.17–100.75)
Interferon alpha-2	IFNα2		50.37 ± 15.50	45.15 (39.78–53.19)
Interferon-gamma	IFNγ		10.13 ± 13.29	6.54 (4.14–9.90)
Growth-Regulated Oncogene	GRO		3443.66 ± 1756.35	2723.24 (1971.57–4599.71)
Interleukin-10	IL-10		6.75 ± 18.75	0.00 (0.00–1.83)
Monocyte Chemotactic Protein-3	MCP-3	CCL-7	44.96 ± 71.85	24.82 (23.81–26.59)
Interleukin-12P40	IL-12P40		13.40 ± 58.07	0.00 (0.00–4.05)
Macrophage-Derived Chemokine	MDC	CCL-22	375.05 ± 224.42	337.02 (167.47–558.62)
Interleukin-12P70	IL-12P70		1.73 ± 3.86	0.00 (0.00–1.64)
Interleukin-13	IL-13		16.88 ± 67.24	0.00 (0.00–0.00)
Interleukin-15	IL-15		6.32 ± 7.91	5.01 (3.48–6.21)
Soluble CD40L	sCD40L		1182.63 ± 1696.64	682.06 (440.57–1147.05)
Interleukin-17a	IL-17a		5.74 ± 3.77	4.87 (3.94–5.71)
Interleukin-1 receptor antagonist	IL-1Ra	IL-1RN	45.26 ± 172.28	0.00 (0.00–0.00)
Interleukin-1alpha	IL-1α		29.00 ± 38.73	18.28 (6.50–31.80)
Interleukin-9	IL-9		0.84 ± 4.90	0.00 (0.00–0.00)
Interleukin-1beta	IL-1β		3.44 ± 1.39	3.06 (2.83–3.62)
Interleukin-2	IL-2		2.11 ± 5.91	1.07 (0.63–1.36)
Interleukin-3	IL-3		5072.64 ± 6.82	5074.16 (5072.73–5074.96)
Interleukin-4	IL-4		0.87 ± 4.94	0.00 (0.00–0.00)
Interleukin-5	IL-5		2.50 ± 7.82	0.00 (0.00–0.00)
Interleukin-6	IL-6		15.55 ± 10.10	12.63 (10.26–17.56)
Interleukin-7	IL-7		22.08 ± 0.89	21.73 (21.55–22.38)
Interleukin-8	IL-8		2321.01 ± 3161.59	111.69 (39.98–5725.30)
Interferon gamma-induced protein-10	IP-10	CXCL-10	860.20 ± 355.13	860.67 (633.77–1019.03)
Monocyte chemoattractant protein-1	MCP-1	CCL-2	837.05 ± 375.51	820.91 (550.17–1053.62)
Macrophage inflammatory protein-1 alpha	MIP-1α	CCL-3	5237.02 ± 450.11	5455.83 (5221.66–5465.85)
Macrophage Inflammatory Protein-1 beta	MIP-1β	CCL-4	98.84 ± 154.98	45.61 (40.44–89.04)
Tumor necrosis factor alpha	TNFα		79.23 ± 141.10	37.27 (12.62–91.28)
Tumor necrosis factor beta	TNFβ	LT-α	41.78 ± 139.08	0.00 (0.00–0.00)
Vascular endothelial growth factor	VEGF	VPF	83.93 ± 33.29	73.62 (68.07–84.73)
Eotaxin-2	Eotaxin-2	CCL-24	517.82 ± 329.27	517.58 (202.47–724.16)
Monocyte chemoattractant protein-2	MCP-2	CCL-8	25.57 ± 2.54	24.59 (23.98–26.21)
B-cell-attracting chemokine-1	BCA-1	CXCL-13	73.33 ± 169.96	30.27 (20.71–50.49)
Monocyte chemoattractant protein-4	MCP-4	CCL-13	73.74 ± 41.99	56.08 (44.66–92.63)
I-309	I-309	CCL-1	7.33 ± 0.35	7.27 (7.16–7.38)
Interleukin-16	IL-16		137.79 ± 159.28	95.07 (91.54–102.78)
Thymus and Activation-Regulated Chemokine	TARC	CCL-17	47.78 ± 52.30	27.90 (15.04–48.97)
6CKINE	6CKINE	CCL-21, SLC	210.98 ± 70.17	192.34 (170.61–228.51)
Eotaxin-3	Eotaxin-3	CCL-26	107.29 ± 66.91	94.03 (86.37–103.47)
Leukemia inhibitory factor	LIF	CDF	4.88 ± 4.38	3.59 (2.82–5.89)
Thrombopoietin	TPO	THPO, MGDF	517.09 ± 1000.06	316.89 (216.15–449.68)
Stem cell factor	SCF	KITLG, KL-1	32.90 ± 22.80	26.78 (21.17–39.88)
Thymic stromal lymphopoietin	TSLP		179.88 ± 1035.22	0.00 (0.00–0.00)
Interleukin-33	IL-33		12.46 ± 43.90	2.77 (0.48–5.44)
Interleukin-20	IL-20		260.67 ± 19.81	255.26 (251.74–260.19)
Interleukin-21	IL-21		0.94 ± 3.57	0.00 (0.00–0.00)
Interleukin-23	IL-23		221.80 ± 725.15	52.66 (0.00–120.77)
TNF-related apoptosis-inducing ligand	TRAIL	TNFSF-10	49.41 ± 31.52	44.92 (34.06–63.94)
Cutaneous T cell-attracting chemokine	CTACK		1153.68 ± 379.68	1154.83 (932.83–1313.16)
Stromal cell-derived factor-1 alpha+beta	SDF-1α+β	CXCL-12	1924.68 ± 675.83	1707.43 (1342.32–2578.24)
Epithelial-derived neutrophil-activating peptide-78	ENA-78	CXCL-5	552.76 ± 688.99	260.71 (175.97–555.67)
Macrophage inflammatory protein-1 delta	MIP-1D	CCL-15	4686.69 ± 2379.29	4700.68 (3348.28–5445.67)
Interleukin-28a	IL-28a		97.18 ± 313.03	16.55 (10.28–35.34)
Soluble CD30	sCD30		67.43 ± 9.44	67.62 (61.09–74.15)
Soluble epidermal growth factor receptor	sEGFR		24,334.40 ± 9315.22	23,139.30 (18,761.40–28,847.50)
Soluble glycoprotein 130	sgp130		44,580.11 ± 9980.83	44,151.70 (37,428.05–52,851.60)
Soluble interleukin-1 receptor I	sIL-1RI	sCD121α	61.04 ± 7.57	60.08 (56.97–64.75)
Soluble interleukin-1 receptor II	sIL-1RII		1304.63 ± 926.28	1090.63 (713.99–1496.65)
Soluble interleukin-2 receptor alpha	sIL-2Rα	sCD25	257.57 ± 120.89	237.76 (172.90–280.88)
Soluble interleukin-4 receptor	sIL-4R		245.62 ± 8.14	244.34 (240.11–248.56)
Soluble interleukin-6 receptor	sIL-6R		6560.42 ± 2361.00	6403.40 (4880.51–7926.86)
Soluble receptor for advanced glycation endproducts	sRAGE		74.29 ± 37.36	71.36 (57.67–75.93)
Soluble tumor necrosis factor receptor type I	sTNFRI		258.07 ± 182.50	201.49 (146.74–284.48)
Soluble tumor necrosis factor receptor type II	sTNFRII		2184.49 ± 1149.96	1811.82 (1378.17–2756.11)
Soluble vascular endothelial growth factor receptor 1	sVEGFR-1		71,122.51 ± 140,802.70	516.84 (457.70–576.02)
Soluble vascular endothelial growth factor receptor 2	sVEGFR-2		1760.73 ± 636.97	1658.82 (1361.15–2116.92)
Soluble vascular endothelial growth factor receptor 3	sVEGFR-3		338.00 ± 107.60	339.94 (251.26–384.29)
Soluble hepatocyte growth factor receptor/cMET	sHGFR/cMET		6807.43 ± 1489.53	6838.36 (5674.58–7638.24)
Soluble AXL	sAXL		588.95 ± 260.73	499.09 (407.31–779.31)
Osteopontin	OPN		354.57 ± 278.07	342.54 (151.24–513.54)
Soluble platelet endothelial cell adhesion molecule 1	sPECAM-1		166.62 ± 44.54	165.49 (137.77–192.28)
Soluble HER2	sHER-2		185.20 ± 56.64	186.93 (140.20–220.25)
Soluble HER3	sHER-3		179.39 ± 108.46	160.54 (88.78–229.25)
Soluble urokinase plasminogen activator receptor	suPAR		91.22 ± 59.12	73.26 (51.61–128.48)
Soluble Tie2	sTie-2		390.80 ± 233.51	327.69 (214.34–545.85)
Soluble interleukin-6 receptor alpha	sIL-6Rα		1049.87 ± 257.05	1037.61 (904.18–1225.98)
Soluble neuropilin-1	sNRP-1		1189.92 ± 181.46	1189.70 (1043.70–1319.07)
Soluble E-selectin	sE-selectin		180.52 ± 127.95	138.37 (90.08–224.65)
Platelet-derived growth factor-AB/BB	PDGF-AB/BB		272.10 ± 471.92	108.76 (47.73–266.30)
Thrombospondin-2	THBS-2		245.45 ± 269.39	181.85 (83.30–270.85)

**Table 2:** (**a**) Longitudinal analysis of the trajectories of the six key biomarkers in progressive disease (PD) patients. Values are median plasma concentrations (pg/mL). (**b**) Longitudinal analysis of the trajectories of the six key biomarkers in stable disease (SD) patients. Values are median plasma concentrations (pg/mL).

(**a**)					
**Biomarker**	**Day 0**	**Day 28**	**Day 56**	**Day 84**	**Day 112**
EOTAXIN-1	326.25	338.58	370.41	399.80	386.73
MCP-4	52.06	55.10	57.28	74.74	80.39
IL-16	93.52	96.17	94.07	95.07	94.41
TRAIL	42.20	43.68	40.48	43.19	50.60
PDGF-AB/BB	63.64	116.70	61.43	69.06	82.40
THBS-2	197.60	206.05	294.55	217.30	450.85
(**b**)					
**Biomarker**	**Day 0**	**Day 28**	**Day 56**	**Day 84**	**Day 112**
EOTAXIN-1	740.96	734.50	737.80	647.06	611.60
MCP-4	76.46	82.58	76.46	67.30	70.57
IL-16	105.43	106.75	135.03	112.93	102.56
TRAIL	60.73	63.20	56.77	55.04	47.14
PDGF-AB/BB	184.75	194.92	139.54	161.35	100.88
THBS-2	110.40	76.45	143.65	161.05	184.00

**Table 3:** Three optimal 3-biomarker combinations for risk stratification identified through systematic combinatorial analysis. Risk groups were determined using composite risk scores constructed from standardized biomarker values with optimized directional weights. All three combinations achieved statistically significant risk stratification (*p* < 0.05). Median survival values are reported as high-risk vs. low-risk. HR: Hazard Ratio; CI: Confidence Interval; *p*-values from log-rank test.

Combination	PFS (Months)	PFS *p*	PFS HR (95% CI)	OS (Months)	OS *p*	OS HR (95% CI)	No.
IL-16 + MCP-4 + THBS-2	2.0 vs. 4.0	0.0046	3.24 (1.41–7.46)	5.8 vs. 11.1	0.0010	4.19 (1.68–10.42)	1
MCP-4 + PDGF-AB/BB + THBS-2	2.0 vs. 4.0	0.0014	3.97 (1.66–9.52)	5.5 vs. 11.1	<0.001	6.24 (2.32–16.74)	2
EOTAXIN-1 + IL-16 + THBS-2	2.0 vs. 4.0	0.0011	3.53 (1.58–7.87)	5.5 vs. 9.6	0.0131	2.59 (1.20–5.56)	3

**Table 4:** Univariate and multivariate Cox proportional hazards regression for progression-free survival (PFS). Full cohort (n = 38). ECOG PS: Eastern Cooperative Oncology Group Performance Status. HR: Hazard Ratio. CI: Confidence Interval. CRC: Colorectal cancer. * *p* < 0.05. Note: Data on prior therapy lines and metastatic disease burden were not available from the COMET trial database and could not be included as covariates.

Variable	Comparison	Univariate HR	95% CI	*p*-Value	Multivariate HR	Multiv. *p*
ECOG PS	≥1 vs. 0	3.37	1.13–10.02	0.029 *	3.65	0.021 *
Tumor type	CRC vs. non-CRC	1.30	0.44–3.84	0.634	1.66	0.367

## Data Availability

Individual participant data from the COMET trial (EudraCT: 2007-000065-38) are not publicly available due to privacy and ethical considerations under the European Union General Data Protection Regulation (GDPR). De-identified datasets generated and analyzed during the current study may be made available from the corresponding author upon reasonable request, subject to approval by the institutional review board and execution of appropriate data sharing agreements. The custom R code implementing the Contal and O’Quigley cutpoint algorithm and Manciu’s combinatorial method may similarly be made available to qualified researchers upon reasonable request from the corresponding author.

## Supplementary Materials

The following supporting information can be downloaded at: https://www.mdpi.com/article/10.3390/cancers18111762/s1, Table S1: Biomarker measurements at Day 28—complete plasma concentrations (pg/mL) for all 88 circulating cytokines in all evaluable patients. Table S2: Biomarker measurements at Day 56—complete plasma concentrations (pg/mL) for all 88 circulating cytokines in all evaluable patients. Table S3: Biomarker measurements at Day 84—complete plasma concentrations (pg/mL) for all 88 circulating cytokines in all evaluable patients. Table S4: Biomarker measurements at Day 112—complete plasma concentrations (pg/mL) for all 88 circulating cytokines in all evaluable patients. Table S5: Baseline (Day 0) plasma concentrations (pg/mL) for all 88 circulating cytokines in all evaluable patients. Table S6: Exploratory subgroup analysis restricted to colorectal cancer patients (n = 30)—Cox proportional hazards regression for PFS, patient characteristics, and survival by ECOG PS. Table S7: Assay sensitivities and precision (intra-assay and inter-assay %CV) for all 88 cytokines measured by Luminex^®^ multiplex immunoassay. Table S8: Accuracy and spike recovery data for all 88 cytokines. Figure S1: Kaplan–Meier survival curves for subgroup analyses by ECOG PS (full cohort and CRC subgroup) and by tumor type (CRC vs. non-CRC).

## References

[1] GBD 2023 Cancer Collaborators (2025). The global, regional, and national burden of cancer, 1990–2023. Lancet.

[2] Piotrowski M., Suska K., Jacenik D., Fichna J. (2025). Current advances in colorectal cancer treatment: A review of recent clinical trials. Expert Opin. Pharmacother..

[3] El Darsa H., El Sayed R., Abdel-Rahman O. (2021). What is the real value of metronomic chemotherapy in the treatment of gastrointestinal cancer?. Expert Opin. Pharmacother..

[4] Filippi R., Lombardi P., Depetris I., Fenocchio E., Quarà V., Chilà G., Aglietta M., Leone F. (2018). Rationale for the use of metronomic chemotherapy in gastrointestinal cancer. Expert Opin. Pharmacother..

[5] Shaham S.H., Vij P., Tripathi M.K. (2025). Advances in Targeted and Chemotherapeutic Strategies for Colorectal Cancer: Current Insights and Future Directions. Biomedicines.

[6] Sullo F., Gallio C., Matteucci L., Bittoni A., Muratore M., Esposito L., Ceredi B., Gallo G., Ulivi P., Rapposelli I.G. (2025). Personalized therapy in metastatic colorectal cancer: Biomarker-driven use of biologics. Expert Opin. Biol. Ther..

[7] Banchi M., Fini E., Crucitta S., Bocci G. (2022). Metronomic Chemotherapy in Pediatric Oncology: From Preclinical Evidence to Clinical Studies. J. Clin. Med..

[8] Bandini A., Calabrò P.F., Banchi M., Orlandi P., Bocci G. (2024). Metronomic Chemotherapy in Elderly Patients. Curr. Oncol. Rep..

[9] Natale G., Bocci G. (2018). Does metronomic chemotherapy induce tumor angiogenic dormancy? A review of available preclinical and clinical data. Cancer Lett..

[10] Bocci G., Kerbel R.S. (2016). Pharmacokinetics of metronomic chemotherapy: A neglected but crucial aspect. Nat. Rev. Clin. Oncol..

[11] Banchi M., Cox M.C., Bocci G. (2024). Metronomic chemotherapy in hematology: Lessons from preclinical and clinical studies to build a solid rationale for future schedules. Cancer Lett..

[12] Gupta N., Verma N., Patel B. (2024). Efficacy and Safety of Metronomic Capecitabine in Hepatocellular Carcinoma: A Systematic Review and Meta-analysis. J. Gastrointest. Cancer.

[13] Chen L.B., Cao X.B., Li J.M., Liu C.M., Jiang T.M. (2022). Efficacy and safety of metronomic chemotherapy in maintenance therapy for metastatic colorectal cancer: A systematic review of randomized controlled trials. Medicine.

[14] Hong J.H., Woo I.S. (2023). Metronomic chemotherapy as a potential partner of immune checkpoint inhibitors for metastatic colorectal cancer treatment. Cancer Lett..

[15] Valenzuela P., Oaxaca D., Di Desidero T., Parra K., Rodriguez G., Manciu M., Allegrini G., Falcone A., Bocci G., Kirken R.A. (2020). Pharmacodynamic biomarkers in metronomic chemotherapy: Multiplex cytokine measurements in gastrointestinal cancer patients. Clin. Exp. Med..

[16] Manciu M., Hosseini S., Di Desidero T., Allegrini G., Falcone A., Bocci G., Kirken R.A., Francia G. (2018). Optimization of Biomarkers-Based Classification Scores as Progression-Free Survival Predictors: An Intuitive Graphical Representation. Futur. Sci. OA.

[17] Allegrini G., Falcone A., Fioravanti A., Barletta M.T., Orlandi P., Loupakis F., Cerri E., Masi G., Di Paolo A., Kerbel R.S. (2008). A pharmacokinetic and pharmacodynamic study on metronomic irinotecan in metastatic colorectal cancer patients. Br. J. Cancer.

[18] Allegrini G., Di Desidero T., Barletta M.T., Fioravanti A., Orlandi P., Canu B., Chericoni S., Loupakis F., Di Paolo A., Masi G. (2012). Clinical, pharmacokinetic and pharmacodynamic evaluations of metronomic UFT and cyclophosphamide plus celecoxib in patients with advanced refractory gastrointestinal cancers. Angiogenesis.

[19] Spehner L., Bouard A., El Kaddissi A., Kroemer M., Kim S., Nguyen T., Klajer E., Fein F., Abdeljaoued S., Loyon R. (2025). Clinical and biological determinants of multimodal metronomic chemotherapy efficacy in chemo-refractory gastrointestinal cancers. Int. J. Cancer.

[20] Steup C., Kennel K.B., Neurath M.F., Fichtner-Feigl S., Greten F.R. (2025). Current and emerging concepts for systemic treatment of metastatic colorectal cancer. Gut.

[21] Wold S., Sjöström M., Eriksson L. (2001). PLS-regression: A basic tool of chemometrics. Chemom. Intell. Lab. Syst..

[22] Contal C., O’Quigley J. (1999). An application of changepoint methods in studying the effect of age on survival in breast cancer. Comput. Stat. Data Anal..

[23] Kaplan E.L., Meier P. (1958). Nonparametric estimation from incomplete observations. J. Am. Stat. Assoc..

[24] Cox D.R. (1972). Regression models and life-tables. J. R. Stat. Soc. Ser. B.

[25] Harrell F.E., Lee K.L., Mark D.B. (1996). Multivariable prognostic models. Stat. Med..

[26] Akaike H. (1974). A new look at the statistical model identification. IEEE Trans. Autom. Control.

[27] Efron B., Tibshirani R.J. (1993). An Introduction to the Bootstrap.

[28] R Core Team (2025). R: A Language and Environment for Statistical Computing.

[29] Niewold T.B., Lehman J.S., Gunnarsson I., Meves A., Oke V. (2025). Role of interleukin-16 in human diseases: A novel potential therapeutic target. Front. Immunol..

[30] Iwamoto T., Okamoto H., Kobayashi S., Ikari K., Toyama Y., Tomatsu T., Kamatani N., Momohara S. (2007). A role of monocyte chemoattractant protein-4 (MCP-4)/CCL13 from chondrocytes in rheumatoid arthritis. FEBS J..

[31] Zhao X., Zhou B., Cai J., Shen H., Dai C., Wang M. (2025). Decoding the roles of CCL11 in human malignancies and clinical implications. Biochem. Pharmacol..

[32] Calabro N.E., Kristofik N.J., Kyriakides T.R. (2014). Thrombospondin-2 and extracellular matrix assembly. Biochim. Biophys. Acta (BBA) —Gen. Subj..

[33] Irma J., Kartasasmita A.S., Kartiwa A., Irfani I., Rizki S.A., Onasis S. (2025). From Growth Factors to Structure: PDGF and TGF-β in Granulation Tissue Formation. J. Cell. Mol. Med..

[34] Zhang T., Guo Y., Qiu B., Dai X., Wang Y., Cao X. (2025). Global, regional, and national trends in colorectal cancer burden from 1990 to 2021 and projections to 2040. Front. Oncol..

[35] Siegel R.L., Wagle N.S., Cercek A., Smith R.A., Jemal A. (2023). Colorectal cancer statistics, 2023. CA Cancer J. Clin..

[36] Woo I.S., Jung Y.H. (2017). Metronomic chemotherapy in metastatic colorectal cancer. Cancer Lett..

[37] Cheng O.J., Lebish E.J., Jensen O., Jacenik D., Trivedi S., Cacioppo J.G., Aubé J., Beswick E.J., Leung D.T. (2024). Mucosal-associated invariant T cells modulate innate immune cells and inhibit colon cancer growth. Scand. J. Immunol..

[38] Bailey C., Negus R., Morris A., Ziprin P., Goldin R., Allavena P., Peck D., Darzi A. (2007). Chemokine expression is associated with the accumulation of tumour associated macrophages (TAMs) and progression in human colorectal cancer. Clin. Exp. Metastasis.

[39] Manzat Saplacan R.M., Balacescu L., Gherman C., Chira R.I., Craiu A., Mircea P.A., Lisencu C., Balacescu O. (2017). The Role of PDGFs and PDGFRs in Colorectal Cancer. Mediat. Inflamm..

[40] Qu H., Hasen G., Hou Y., Zhang C. (2022). THBS2 promotes cell migration and invasion in colorectal cancer via modulating Wnt/β-catenin signaling pathway. Kaohsiung J. Med. Sci..

[41] Bocci G., Francia G., Man S., Lawler J., Kerbel R.S. (2003). Thrombospondin 1, a mediator of the antiangiogenic effects of low-dose metronomic chemotherapy. Proc. Natl. Acad. Sci. USA.

[42] Han C.J., Ning X., Burd C.E., Spakowicz D.J., Tounkara F., Kalady M.F., Noonan A.M., McCabe S., Von Ah D. (2024). Chemotoxicity and Associated Risk Factors in Colorectal Cancer: A Systematic Review and Meta-Analysis. Cancers.

[43] Gupta A.K. (2026). Metronomic chemotherapy in resource-limited settings: Evidence, opportunities, and future directions. Med. Oncol..

[44] Schmitt M., Greten F.R. (2021). The inflammatory pathogenesis of colorectal cancer. Nat. Rev. Immunol..

[45] Zafari N., Khosravi F., Rezaee Z., Esfandyari S., Bahiraei M., Bahramy A., Ferns G.A., Avan A. (2022). The role of the tumor microenvironment in colorectal cancer and the potential therapeutic approaches. J. Clin. Lab. Anal..

[46] Kerbel R.S., Grothey A. (2015). Gastrointestinal cancer: Rationale for metronomic chemotherapy in phase III trials. Nat. Rev. Clin. Oncol..

