# Supplementary material for: Prognostic Circulating Cytokine Panels for Metronomic Chemotherapy in Metastatic Gastrointestinal Cancer: Exploratory Pharmacodynamic Biomarker Analysis of the Phase II COMET Trial
Source: Cancers (Basel). 2026 May 28;18(11):1762. doi: 10.3390/cancers18111762 (PMC13255639; doi:10.3390/cancers18111762)
Supplement: Supplementary file 1 [file cancers-18-01762-s001.zip › cancers-4290816-Luminex Analysis.pdf]

Luminex Analysis

Peripheral blood samples were collected at baseline and at days 28, 56, 84, and 112 of treatment. Blood was collected in plasma vacutainer tubes (Becton Dickinson Vacutainer System, Rutherford, NJ, USA), temporarily stored for a maximum of 10 min at 4°C, and then centrifuged at 4000 rpm for 10 min at 4°C to separate plasma. Plasma samples were aliquoted and stored at -80°C until analysis. Twenty-five microliters of plasma from 34 patients were evaluated by Luminex® (HCYTOMAG-60K, HCYP2MAG-62K, HSCRMAG-32K and HANG2MAG-12K purchased from Merck Millipore, Darmstadt, Germany). Samples were loaded into the 96-well plate supplied with the kit, and an equal volume of a premix of 88 **Luminex beads** was added to each well. The plate was incubated overnight at 4°C with gentle shaking. The beads were designed to measure the following cytokines: EGF, Eotaxin, G-CSF, GM-CSF, IFNα2, IFNγ, IL-10, IL-12P40, IL-12P70, IL-13, IL-15, IL-17A, IL-1RA, IL-1α, IL-1β, IL-2, IL-3, IL-4, IL-5, IL-6, IL-7, IL-8, IP-10, MCP-1, MIP-1α, MIP-1β, TNFα, TNFβ, VEGF,FGF-2, TGF-α, FIT-3L, Fractalkine, GRO, MCP-3, MDC, sCD40L, IL-9, Eotaxin-2/CCL24, MCP-2, BCA-1/CXCL13, MCP-4, I-309/CCL1, IL-16, TARC/CCL17, 6Ckine/CCL21/Exodus-2, Eotaxin-3/CCL26, LIF, TPO, SCF, TSLP, IL-33/NF-HEV (mature), IL-20, IL-21, IL-23, TRAIL/TNFSF10, CTACK/CCL27, SDF-1α+β/CXCL12, ENA-78/CXCL5, MIP-1δ/MIP-5/CCL15, IL-28A/IFNλ2, soluble CD30 (sCD30, sTNFRSF8), soluble Epidermal Growth Factor Receptor (sEGFR), soluble gp130 (sgp130), soluble Interleukin-1 Receptor Type I (sIL-1RI, sCD121a), soluble Interleukin-1 Receptor Type II (sIL-1RII, sCD121b), soluble Interleukin-2 Receptor alpha (sIL-2Rα, CD25), soluble Interleukin-4 Receptor (sIL-4R, CD124), soluble Interleukin-6 Receptor (sIL-6R, CD126), soluble Receptor for Advanced Glycation Endproducts (sRAGE), soluble Tumor Necrosis Factor Receptor I (sTNFRI, TNFRSF1A), soluble Tumor Necrosis Factor Receptor II (sTNFRII, TNFRSF1B), soluble Vascular Endothelial Growth Factor Receptor 1 (sVEGFR1, sFlt-1), soluble Vascular Endothelial Growth Factor Receptor 2 (sVEGFR2, sFlk-1, sKDR), soluble Vascular Endothelial Growth Factor Receptor 3 (sVEGFR3, sFlt-4), soluble E-Selectin, (sE-Selectin), Osteopontin (OPN), Platelet Derived Growth Factor-AB/BB (PDGF-AB/BB), soluble Platelet Endothelial Cell Adhesion Molecule-1 (sPECAM-1), Thrombospondin-2 (TSP-2), soluble AXL (sAXL), soluble Human Epidermal Growth Factor Receptor 2 (sHer2), soluble Human Epidermal Growth Factor Receptor 3 (sHer3), soluble Hepatocyte Growth Factor Receptor/c-Met (sHGFR/c-Met), soluble IL-6Rα, soluble Neuropilin-1 (sNRP-1), soluble Tie-2 (sTie-2), soluble Urokinase-type Plasminogen Activator Receptor (suPAR).

After overnight incubation, the beads were washed and incubated with 25 µL of secondary biotinylated detection antibody for 1 h at room temperature, according to the manufacturer's instructions. Beads were then mixed with 25 µL of streptavidin-PE conjugate, washed, and resuspended in 100 µL of sheath fluid. Samples were analyzed on a FlexMap3D instrument (Merck Millipore) using xPONENT® software (Merck Millipore), and cytokine concentrations were calculated using Belysa Immunoassay Curve-Fitting software (Sigma-Aldrich, St. Louis, USA), following the manufacturer's protocols and settings. Minimum detectable concentrations, assay sensitivities and precision of the analysis are reported in Supplementary Table S7, including intra-assay and inter-assay %CV's. The accuracy with spike recovery is reported in Supplementary Table S8.
