# Supplementary material for: Prognostic Circulating Cytokine Panels for Metronomic Chemotherapy in Metastatic Gastrointestinal Cancer: Exploratory Pharmacodynamic Biomarker Analysis of the Phase II COMET Trial
Source: Cancers (Basel). 2026 May 28;18(11):1762. doi: 10.3390/cancers18111762 (PMC13255639; doi:10.3390/cancers18111762)

Supplementary Table S1. Biomarker measurements at Day 28 — plasma concentrations (pg/ml) for all 88 circulating cytokines.

**Supplementary Table S1. Biomarker measurements at Day 28. Complete plasma concentrations (pg/ml) for all 88 circulating cytokines. Values are mean ± standard deviation (SD) and percentiles. n = 30 (PD: 17, SD: 13). IQR: interquartile range (25th–75th percentile).**						
Analyzed circulating protein	Acronym	Mean (pg/ml) ± SD	P25	Median	P75	IQR
Epidermal growth factor	EGF	41.35 ± 34.52	24.09	37.89	55.15	31.07
Fibroblast growth factor-2	FGF-2	494.78 ± 301.61	343.97	464.62	615.42	271.45
Eotaxin-1	Eotaxin-1	274.27 ± 174.68	186.93	256.80	344.14	157.21
Transforming growth factor alpha	TGFα	371.10 ± 340.27	200.97	337.08	507.21	306.24
Granulocyte colony-stimulating factor	G-CSF	301.37 ± 355.16	123.79	265.85	443.43	319.64
Fms-like tyrosine kinase 3 ligand	FLT-3L	352.94 ± 214.78	245.55	331.46	438.86	193.30
Granulocyte-Macrophage Colony-Stimulating Factor	GM-CSF	193.07 ± 168.03	109.06	176.27	260.29	151.23
Fractalkine	Fractalkine	347.55 ± 173.70	260.70	330.18	417.03	156.33
Interferon alpha-2	IFNα2	405.47 ± 284.77	263.08	376.99	519.37	256.29
Interferon-gamma	IFNγ	387.34 ± 147.95	313.36	372.54	446.52	133.15
Growth-Regulated Oncogene	GRO	162.21 ± 51.26	136.58	157.09	182.72	46.13
Interleukin-10	IL-10	211.39 ± 111.74	155.51	200.21	256.08	100.57
Monocyte Chemotactic Protein-3	MCP-3	176.72 ± 88.11	132.67	167.91	211.97	79.30
Interleukin-12P40	IL-12P40	426.57 ± 371.84	240.65	389.39	575.31	334.65
Macrophage-Derived Chemokine	MDC	178.15 ± 133.20	111.55	164.83	231.43	119.88
Interleukin-12P70	IL-12P70	96.46 ± 110.74	41.09	85.38	140.75	99.66
Interleukin-13	IL-13	453.20 ± 369.01	268.69	416.30	600.80	332.10
Interleukin-15	IL-15	80.93 ± 93.70	34.08	71.56	118.41	84.33
Soluble CD40L	sCD40L	112.24 ± 39.23	92.63	108.32	127.93	35.30
Interleukin-17a	IL-17a	212.15 ± 135.70	144.30	198.58	266.43	122.13
Interleukin-1 receptor antagonist	IL-1Ra	425.84 ± 183.91	333.88	407.45	499.41	165.52
Interleukin-1alpha	IL-1α	35.17 ± 24.52	22.90	32.71	44.98	22.07
Interleukin-9	IL-9	25.48 ± 22.85	14.06	23.20	34.62	20.56
Interleukin-1beta	IL-1β	246.54 ± 267.94	112.58	219.75	353.72	241.14
Interleukin-2	IL-2	468.00 ± 239.07	348.47	444.10	563.63	215.16

Interleukin-3	IL-3	394.52 ± 295.68	246.68	364.95	512.80	266.12
Interleukin-4	IL-4	475.47 ± 176.63	387.16	457.81	546.13	158.97
Interleukin-5	IL-5	230.96 ± 273.05	94.43	203.65	340.18	245.75
Interleukin-6	IL-6	32.65 ± 18.74	23.28	30.77	40.14	16.87
Interleukin-7	IL-7	158.28 ± 99.03	108.77	148.38	197.90	89.13
Interleukin-8	IL-8	496.34 ± 234.21	379.23	472.92	590.02	210.78
Interferon gamma-induced protein-10	IP-10	412.74 ± 222.93	301.27	390.45	501.91	200.64
Monocyte chemoattractant protein-1	MCP-1	437.88 ± 189.20	343.28	418.96	513.56	170.28
Macrophage inflammatory protein-1 alpha	MIP-1α	54.74 ± 65.00	22.24	48.24	80.74	58.50
Macrophage Inflammatory Protein-1 beta	MIP-1β	11.55 ± 10.14	6.48	10.54	15.61	9.13
Tumor necrosis factor alpha	TNFα	105.86 ± 86.89	62.42	97.17	140.61	78.20
Tumor necrosis factor beta	TNFβ	162.27 ± 139.84	92.35	148.29	218.21	125.86
Vascular endothelial growth factor	VEGF	340.54 ± 331.95	174.56	307.34	473.32	298.75
Eotaxin-2	Eotaxin-2	65.26 ± 68.28	31.12	58.43	92.57	61.46
Monocyte chemoattractant protein-2	MCP-2	123.18 ± 102.74	71.81	112.91	164.28	92.46
B-cell-attracting chemokine-1	BCA-1	405.74 ± 403.55	203.96	365.39	567.16	363.20
Monocyte chemoattractant protein-4	MCP-4	267.67 ± 200.18	167.58	247.65	347.74	180.16
I-309	I-309	81.31 ± 88.75	36.94	72.44	116.81	79.87
Interleukin-16	IL-16	299.39 ± 297.72	150.53	269.62	418.48	267.95
Thymus and Activation-Regulated Chemokine	TARC	483.31 ± 449.61	258.50	438.35	663.15	404.65
6CKINE	6CKINE	153.59 ± 112.50	97.34	142.34	198.59	101.25
Eotaxin-3	Eotaxin-3	90.46 ± 49.71	65.60	85.49	110.34	44.74
Leukemia inhibitory factor	LIF	429.38 ± 387.07	235.84	390.67	584.20	348.36
Thrombopoietin	TPO	87.64 ± 67.44	53.92	80.90	114.62	60.69
Stem cell factor	SCF	207.39 ± 214.56	100.12	185.94	293.22	193.10
Thymic stromal lymphopoietin	TSLP	416.65 ± 257.39	287.96	390.91	519.60	231.65
Interleukin-33	IL-33	46.22 ± 21.69	35.37	44.05	54.90	19.52
Interleukin-20	IL-20	284.38 ± 178.54	195.11	266.53	355.80	160.68
Interleukin-21	IL-21	22.44 ± 21.78	11.56	20.27	31.15	19.60
Interleukin-23	IL-23	309.19 ± 183.58	217.41	290.84	382.62	165.22
TNF-related apoptosis-inducing ligand	TRAIL	19.98 ± 12.37	13.80	18.75	24.93	11.14
Cutaneous T cell-attracting chemokine	CTACK	295.00 ± 105.35	242.32	284.46	337.14	94.81
Stromal cell-derived factor-1 alpha+beta	SDF-1α+β	58.01 ± 19.56	48.24	56.06	65.84	17.60
Epithelial-derived neutrophil-activating peptide-78	ENA-78	20.53 ± 17.47	11.80	18.78	27.52	15.72
Macrophage inflammatory protein-1 delta	MIP-1D	358.21 ± 313.26	201.58	326.88	483.51	281.93
Interleukin-28a	IL-28a	162.66 ± 100.70	112.31	152.59	202.94	90.63
Soluble CD30	sCD30	10.76 ± 11.04	5.24	9.65	15.17	9.93
Soluble epidermal growth factor receptor	sEGFR	266.60 ± 125.54	203.83	254.05	316.82	112.99
Soluble glycoprotein 130	sgp130	61.61 ± 33.88	44.66	58.22	75.16	30.49
Soluble	sIL-1RI	195.38 ± 181.66	104.55	177.22	268.05	163.50

interleukin-1 receptor I						
Soluble interleukin-1 receptor II	sIL-1RII	40.79 ± 40.80	20.39	36.71	57.10	36.72
Soluble interleukin-2 receptor alpha	sIL-2Rα	121.93 ± 53.48	95.19	116.58	143.32	48.13
Soluble interleukin-4 receptor	sIL-4R	314.04 ± 136.06	246.01	300.43	368.46	122.45
Soluble interleukin-6 receptor	sIL-6R	493.55 ± 449.15	268.98	448.64	673.21	404.24
Soluble receptor for advanced glycation endproducts	sRAGE	367.44 ± 291.57	221.65	338.28	484.06	262.41
Soluble tumor necrosis factor receptor type I	sTNFRI	439.72 ± 469.62	204.91	392.76	627.56	422.66
Soluble tumor necrosis factor receptor type II	sTNFRII	277.29 ± 118.70	217.94	265.42	324.77	106.83
Soluble vascular endothelial growth factor receptor 1	sVEGFR-1	334.50 ± 399.72	134.64	294.53	494.39	359.75
Soluble vascular endothelial growth factor receptor 2	sVEGFR-2	125.93 ± 117.05	67.40	114.22	172.75	105.35
Soluble vascular endothelial growth factor receptor 3	sVEGFR-3	60.16 ± 62.71	28.80	53.89	85.24	56.44
Soluble hepatocyte growth factor receptor/cMET	sHGFR/cMET	271.10 ± 218.16	162.02	249.29	358.36	196.34
Soluble AXL	sAXL	294.52 ± 227.91	180.57	271.73	385.69	205.12
Osteopontin	OPN	332.95 ± 284.47	190.71	304.50	446.73	256.02
Soluble platelet endothelial cell adhesion molecule 1	sPECAM-1	149.76 ± 162.08	68.72	133.55	214.60	145.88
Soluble HER2	sHER-2	67.22 ± 65.22	34.61	60.70	93.30	58.70
Soluble HER3	sHER-3	215.00 ± 84.16	172.92	206.58	248.67	75.75
Soluble urokinase plasminogen activator receptor	suPAR	141.93 ± 105.32	89.27	131.40	184.06	94.79
Soluble Tie2	sTie-2	71.97 ± 62.18	40.88	65.75	96.84	55.96
Soluble interleukin-6 receptor alpha	sIL-6Rα	476.02 ± 450.69	250.68	430.95	656.30	405.62
Soluble neuropilin-1	sNRP-1	278.59 ± 232.45	162.37	255.35	371.58	209.21
Soluble E-selectin	sE-selectin	246.45 ± 243.75	124.58	222.07	343.95	219.37
Platelet-derived growth factor-AB/BB	PDGF-AB/BB	351.47 ± 266.54	218.20	324.82	458.09	239.89
Thrombospondin-2	THBS-2	374.94 ± 330.53	209.67	341.88	507.15	297.47

Supplementary Table S2. Biomarker measurements at Day 56 — plasma concentrations (pg/ml) for all 88 circulating cytokines.

**Supplementary Table S2. Biomarker measurements at Day 56. Complete plasma concentrations (pg/ml) for all 88 circulating cytokines. Values are mean ± standard deviation (SD) and percentiles. n = 27 (PD: 14, SD: 13). IQR: interquartile range (25th–75th percentile).**						
Analyzed circulating protein	Acronym	Mean (pg/ml) ± SD	P25	Median	P75	IQR
Epidermal growth factor	EGF	343.70 ± 132.76	277.32	330.42	396.80	119.48
Fibroblast growth factor-2	FGF-2	87.62 ± 74.01	50.62	80.22	117.23	66.61
Eotaxin-1	Eotaxin-1	167.38 ± 187.99	73.38	148.58	242.57	169.19
Transforming growth factor alpha	TGFα	395.09 ± 425.99	182.09	352.49	565.48	383.39
Granulocyte colony-stimulating factor	G-CSF	210.19 ± 219.85	100.27	188.21	298.14	197.87
Fms-like tyrosine kinase 3 ligand	FLT-3L	285.69 ± 299.27	136.05	255.76	405.40	269.35
Granulocyte-Macrophage Colony-Stimulating Factor	GM-CSF	163.99 ± 130.11	98.94	150.98	216.04	117.10
Fractalkine	Fractalkine	222.94 ± 149.59	148.15	207.98	282.77	134.63
Interferon alpha-2	IFNα2	199.10 ± 133.97	132.11	185.70	252.69	120.57
Interferon-gamma	IFNγ	77.87 ± 33.34	61.20	74.53	91.21	30.01
Growth-Regulated Oncogene	GRO	158.48 ± 146.49	85.24	143.83	217.08	131.84
Interleukin-10	IL-10	332.14 ± 325.34	169.47	299.60	462.27	292.81
Monocyte Chemotactic Protein-3	MCP-3	364.01 ± 376.69	175.66	326.34	514.68	339.02
Interleukin-12P40	IL-12P40	56.12 ± 36.39	37.92	52.48	70.67	32.75
Macrophage-Derived Chemokine	MDC	312.82 ± 112.24	256.70	301.60	357.72	101.02
Interleukin-12P70	IL-12P70	437.11 ± 415.60	229.31	395.55	603.35	374.04
Interleukin-13	IL-13	189.18 ± 63.68	157.34	182.81	214.65	57.31
Interleukin-15	IL-15	51.41 ± 16.83	43.00	49.73	58.15	15.15
Soluble CD40L	sCD40L	128.36 ± 66.73	94.99	121.68	155.05	60.06
Interleukin-17a	IL-17a	79.49 ± 58.11	50.43	73.68	102.73	52.30
Interleukin-1 receptor antagonist	IL-1Ra	402.54 ± 375.12	214.98	365.03	552.59	337.60
Interleukin-1alpha	IL-1α	318.73 ± 362.67	137.39	282.46	463.79	326.41
Interleukin-9	IL-9	261.59 ± 86.35	218.41	252.95	296.13	77.72
Interleukin-1beta	IL-1β	47.19 ± 19.39	37.49	45.25	54.95	17.45
Interleukin-2	IL-2	201.24 ± 178.14	112.17	183.43	272.49	160.32

Interleukin-3	IL-3	464.47 ± 203.78	362.58	444.10	545.99	183.40
Interleukin-4	IL-4	246.79 ± 217.25	138.17	225.07	333.69	195.53
Interleukin-5	IL-5	237.63 ± 130.64	172.31	224.57	289.89	117.58
Interleukin-6	IL-6	96.94 ± 99.86	47.01	86.95	136.88	89.87
Interleukin-7	IL-7	443.38 ± 219.42	333.67	421.44	531.14	197.47
Interleukin-8	IL-8	140.30 ± 103.32	88.64	129.97	181.63	92.99
Interferon gamma-induced protein-10	IP-10	213.48 ± 192.06	117.44	194.27	290.30	172.86
Monocyte chemoattractant protein-1	MCP-1	37.02 ± 22.88	25.58	34.74	46.17	20.59
Macrophage inflammatory protein-1 alpha	MIP-1α	400.88 ± 326.24	237.76	368.26	531.38	293.61
Macrophage Inflammatory Protein-1 beta	MIP-1β	32.70 ± 33.11	16.14	29.39	45.94	29.80
Tumor necrosis factor alpha	TNFα	360.20 ± 149.17	285.62	345.28	419.86	134.25
Tumor necrosis factor beta	TNFβ	346.34 ± 257.72	217.48	320.57	449.43	231.95
Vascular endothelial growth factor	VEGF	108.01 ± 117.89	49.06	96.22	155.16	106.11
Eotaxin-2	Eotaxin-2	414.96 ± 483.10	173.41	366.65	608.20	434.79
Monocyte chemoattractant protein-2	MCP-2	193.34 ± 218.38	84.15	171.51	280.70	196.54
B-cell-attracting chemokine-1	BCA-1	313.30 ± 219.52	203.54	291.34	401.10	197.57
Monocyte chemoattractant protein-4	MCP-4	34.09 ± 13.76	27.21	32.72	39.59	12.38
I-309	I-309	253.81 ± 89.35	209.14	244.88	289.55	80.41
Interleukin-16	IL-16	408.50 ± 338.22	239.38	374.67	543.78	304.40
Thymus and Activation-Regulated Chemokine	TARC	102.59 ± 86.92	59.13	93.90	137.36	78.23
6CKINE	6CKINE	277.02 ± 168.76	192.64	260.14	344.53	151.89
Eotaxin-3	Eotaxin-3	66.67 ± 75.73	28.80	59.10	96.96	68.16
Leukemia inhibitory factor	LIF	469.22 ± 459.26	239.58	423.29	652.92	413.34
Thrombopoietin	TPO	450.58 ± 161.00	370.08	434.48	514.98	144.90
Stem cell factor	SCF	343.41 ± 395.21	145.80	303.89	501.50	355.69
Thymic stromal lymphopoietin	TSLP	351.06 ± 344.45	178.84	316.62	488.84	310.00
Interleukin-33	IL-33	380.06 ± 450.08	155.02	335.05	560.09	405.07
Interleukin-20	IL-20	245.49 ± 201.52	144.73	225.33	326.09	181.37
Interleukin-21	IL-21	269.89 ± 252.47	143.66	244.65	370.88	227.22
Interleukin-23	IL-23	65.04 ± 42.10	43.99	60.83	81.88	37.89
TNF-related apoptosis-inducing ligand	TRAIL	130.63 ± 130.97	65.15	117.54	183.02	117.87
Cutaneous T cell-attracting chemokine	CTACK	123.65 ± 128.33	59.49	110.82	174.98	115.49
Stromal cell-derived factor-1 alpha+beta	SDF-1α+β	390.86 ± 149.74	315.99	375.89	450.76	134.77
Epithelial-derived neutrophil-activating peptide-78	ENA-78	147.76 ± 137.39	79.07	134.02	202.71	123.65
Macrophage inflammatory protein-1 delta	MIP-1D	41.59 ± 37.55	22.82	37.84	56.61	33.80
Interleukin-28a	IL-28a	142.03 ± 56.76	113.65	136.36	164.74	51.08
Soluble CD30	sCD30	446.48 ± 217.05	337.96	424.78	533.30	195.35
Soluble epidermal growth factor receptor	sEGFR	477.27 ± 394.92	279.81	437.78	635.24	355.43
Soluble glycoprotein 130	sgp130	244.95 ± 194.08	147.91	225.54	322.58	174.67
Soluble	sIL-1RI	287.59 ± 89.48	242.85	278.64	323.38	80.53

interleukin-1 receptor I						
Soluble interleukin-1 receptor II	sIL-1RII	274.12 ± 154.44	196.90	258.68	335.90	139.00
Soluble interleukin-2 receptor alpha	sIL-2Rα	276.51 ± 319.38	116.82	244.57	404.26	287.44
Soluble interleukin-4 receptor	sIL-4R	371.72 ± 271.58	235.94	344.57	480.35	244.42
Soluble interleukin-6 receptor	sIL-6R	331.90 ± 256.27	203.77	306.28	434.41	230.65
Soluble receptor for advanced glycation endproducts	sRAGE	494.32 ± 220.55	384.05	472.27	582.55	198.50
Soluble tumor necrosis factor receptor type I	sTNFRI	361.86 ± 157.66	283.03	346.09	424.92	141.89
Soluble tumor necrosis factor receptor type II	sTNFRII	442.67 ± 392.25	246.54	403.44	599.57	353.02
Soluble vascular endothelial growth factor receptor 1	sVEGFR-1	297.36 ± 354.05	120.34	261.96	438.98	318.64
Soluble vascular endothelial growth factor receptor 2	sVEGFR-2	232.18 ± 150.66	156.85	217.11	292.44	135.59
Soluble vascular endothelial growth factor receptor 3	sVEGFR-3	156.49 ± 103.36	104.81	146.15	197.84	93.03
Soluble hepatocyte growth factor receptor/cMET	sHGFR/cMET	498.99 ± 486.22	255.88	450.37	693.48	437.60
Soluble AXL	sAXL	101.73 ± 63.70	69.88	95.36	127.21	57.33
Osteopontin	OPN	402.97 ± 299.17	253.38	373.05	522.63	269.25
Soluble platelet endothelial cell adhesion molecule 1	sPECAM-1	151.80 ± 65.54	119.03	145.25	178.02	58.98
Soluble HER2	sHER-2	139.59 ± 84.20	97.49	131.17	173.27	75.78
Soluble HER3	sHER-3	463.04 ± 419.49	253.30	421.10	630.84	377.54
Soluble urokinase plasminogen activator receptor	suPAR	449.55 ± 492.41	203.34	400.30	646.51	443.17
Soluble Tie2	sTie-2	189.22 ± 168.01	105.22	172.42	256.42	151.21
Soluble interleukin-6 receptor alpha	sIL-6Rα	357.25 ± 162.38	276.06	341.01	422.20	146.14
Soluble neuropilin-1	sNRP-1	378.52 ± 200.15	278.44	358.50	458.58	180.14
Soluble E-selectin	sE-selectin	16.98 ± 6.42	13.77	16.34	19.55	5.77
Platelet-derived growth factor-AB/BB	PDGF-AB/BB	100.77 ± 111.86	44.83	89.58	145.51	100.68
Thrombospondin-2	THBS-2	151.51 ± 132.23	85.40	138.29	204.41	119.01

Supplementary Table S3. Biomarker measurements at Day 84 — plasma concentrations (pg/ml) for all 88 circulating cytokines.

**Supplementary Table S3. Biomarker measurements at Day 84. Complete plasma concentrations (pg/ml) for all 88 circulating cytokines. Values are mean ± standard deviation (SD) and percentiles. n = 19 (PD: 6, SD: 13). IQR: interquartile range (25th–75th percentile).**						
Analyzed circulating protein	Acronym	Mean (pg/ml) ± SD	P25	Median	P75	IQR
Epidermal growth factor	EGF	249.77 ± 200.07	149.74	229.76	329.80	180.07
Fibroblast growth factor-2	FGF-2	405.82 ± 238.85	286.40	381.94	501.36	214.96
Eotaxin-1	Eotaxin-1	163.88 ± 151.40	88.18	148.74	224.44	136.26
Transforming growth factor alpha	TGFα	245.65 ± 153.14	169.08	230.34	306.91	137.83
Granulocyte colony-stimulating factor	G-CSF	205.72 ± 122.78	144.33	193.44	254.83	110.50
Fms-like tyrosine kinase 3 ligand	FLT-3L	87.96 ± 76.40	49.76	80.32	118.52	68.76
Granulocyte-Macrophage Colony-Stimulating Factor	GM-CSF	447.42 ± 411.60	241.62	406.26	612.06	370.44
Fractalkine	Fractalkine	319.22 ± 192.74	222.85	299.94	396.31	173.47
Interferon alpha-2	IFNα2	219.59 ± 94.37	172.41	210.15	257.34	84.93
Interferon-gamma	IFNγ	365.72 ± 207.30	262.07	344.99	448.64	186.57
Growth-Regulated Oncogene	GRO	149.43 ± 176.72	61.06	131.75	220.11	159.05
Interleukin-10	IL-10	173.47 ± 204.84	71.05	152.98	255.40	184.36
Monocyte Chemotactic Protein-3	MCP-3	310.89 ± 306.24	157.77	280.27	433.38	275.61
Interleukin-12P40	IL-12P40	315.85 ± 145.55	243.08	301.30	374.07	130.99
Macrophage-Derived Chemokine	MDC	357.88 ± 190.71	262.53	338.81	434.17	171.64
Interleukin-12P70	IL-12P70	206.82 ± 86.33	163.65	198.18	241.35	77.70
Interleukin-13	IL-13	33.92 ± 21.40	23.22	31.78	42.48	19.26
Interleukin-15	IL-15	11.97 ± 7.74	8.10	11.20	15.07	6.97
Soluble CD40L	sCD40L	425.82 ± 510.40	170.62	374.78	629.98	459.36
Interleukin-17a	IL-17a	214.35 ± 85.12	171.79	205.84	248.40	76.61
Interleukin-1 receptor antagonist	IL-1Ra	176.35 ± 63.03	144.84	170.05	201.56	56.72
Interleukin-1alpha	IL-1α	112.66 ± 96.80	64.26	102.98	151.38	87.12
Interleukin-9	IL-9	310.33 ± 101.86	259.40	300.15	351.08	91.68
Interleukin-1beta	IL-1β	246.35 ± 167.59	162.55	229.59	313.38	150.83
Interleukin-2	IL-2	201.94 ± 85.12	159.39	193.43	235.99	76.61

Interleukin-3	IL-3	199.71 ± 78.30	160.57	191.89	231.03	70.47
Interleukin-4	IL-4	344.64 ± 304.83	192.22	314.16	466.57	274.35
Interleukin-5	IL-5	316.24 ± 210.85	210.81	295.15	400.58	189.76
Interleukin-6	IL-6	478.61 ± 302.42	327.41	448.37	599.58	272.18
Interleukin-7	IL-7	105.90 ± 67.36	72.22	99.16	132.84	60.62
Interleukin-8	IL-8	456.80 ± 201.93	355.84	436.61	537.57	181.73
Interferon gamma-induced protein-10	IP-10	383.02 ± 438.31	163.86	339.19	558.34	394.48
Monocyte chemoattractant protein-1	MCP-1	426.71 ± 306.82	273.29	396.02	549.44	276.14
Macrophage inflammatory protein-1 alpha	MIP-1α	227.80 ± 247.02	104.29	203.09	326.60	222.32
Macrophage Inflammatory Protein-1 beta	MIP-1β	356.60 ± 365.92	173.64	320.01	502.97	329.33
Tumor necrosis factor alpha	TNFα	93.27 ± 79.04	53.75	85.37	124.89	71.14
Tumor necrosis factor beta	TNFβ	370.42 ± 190.55	275.14	351.36	446.64	171.49
Vascular endothelial growth factor	VEGF	107.62 ± 113.76	50.74	96.24	153.12	102.39
Eotaxin-2	Eotaxin-2	286.23 ± 191.44	190.51	267.09	362.81	172.29
Monocyte chemoattractant protein-2	MCP-2	245.52 ± 130.51	180.27	232.47	297.72	117.46
B-cell-attracting chemokine-1	BCA-1	127.70 ± 50.55	102.43	122.65	147.92	45.50
Monocyte chemoattractant protein-4	MCP-4	203.96 ± 88.12	159.90	195.14	239.20	79.31
I-309	I-309	448.92 ± 520.90	188.47	396.83	657.28	468.81
Interleukin-16	IL-16	297.64 ± 113.48	240.90	286.29	343.03	102.13
Thymus and Activation-Regulated Chemokine	TARC	301.28 ± 132.54	235.01	288.03	354.30	119.28
6CKINE	6CKINE	188.91 ± 78.45	149.69	181.07	220.29	70.60
Eotaxin-3	Eotaxin-3	251.22 ± 92.37	205.03	241.98	288.16	83.13
Leukemia inhibitory factor	LIF	143.49 ± 73.21	106.89	136.17	172.77	65.88
Thrombopoietin	TPO	235.74 ± 268.13	101.67	208.92	342.99	241.32
Stem cell factor	SCF	291.48 ± 292.70	145.13	262.21	408.56	263.43
Thymic stromal lymphopoietin	TSLP	364.04 ± 283.20	222.44	335.72	477.32	254.88
Interleukin-33	IL-33	38.60 ± 13.60	31.80	37.24	44.04	12.24
Interleukin-20	IL-20	71.57 ± 54.72	44.21	66.10	93.46	49.25
Interleukin-21	IL-21	173.11 ± 196.92	74.65	153.42	251.88	177.23
Interleukin-23	IL-23	103.78 ± 43.42	82.07	99.44	121.15	39.08
TNF-related apoptosis-inducing ligand	TRAIL	322.29 ± 272.69	185.94	295.02	431.37	245.42
Cutaneous T cell-attracting chemokine	CTACK	31.26 ± 19.06	21.73	29.36	38.89	17.16
Stromal cell-derived factor-1 alpha+beta	SDF-1α+β	224.95 ± 234.07	107.92	201.55	318.58	210.66
Epithelial-derived neutrophil-activating peptide-78	ENA-78	390.63 ± 424.28	178.49	348.21	560.35	381.85
Macrophage inflammatory protein-1 delta	MIP-1D	270.21 ± 309.18	115.63	239.30	393.89	278.26
Interleukin-28a	IL-28a	237.81 ± 133.20	171.21	224.49	291.09	119.88
Soluble CD30	sCD30	74.27 ± 77.89	35.33	66.48	105.43	70.10
Soluble epidermal growth factor receptor	sEGFR	326.89 ± 279.47	187.16	298.94	438.68	251.52
Soluble glycoprotein 130	sgp130	489.21 ± 425.08	276.67	446.70	659.24	382.57
Soluble	sIL-1RI	158.55 ± 146.72	85.18	143.87	217.23	132.05

interleukin-1 receptor I						
Soluble interleukin-1 receptor II	sIL-1RII	463.37 ± 213.48	356.63	442.02	548.76	192.13
Soluble interleukin-2 receptor alpha	sIL-2Rα	472.08 ± 318.46	312.85	440.23	599.46	286.61
Soluble interleukin-4 receptor	sIL-4R	61.10 ± 46.27	37.97	56.47	79.61	41.64
Soluble interleukin-6 receptor	sIL-6R	391.54 ± 290.59	246.25	362.49	507.78	261.53
Soluble receptor for advanced glycation endproducts	sRAGE	138.29 ± 137.01	69.79	124.59	193.10	123.31
Soluble tumor necrosis factor receptor type I	sTNFRI	55.42 ± 50.11	30.37	50.41	75.47	45.10
Soluble tumor necrosis factor receptor type II	sTNFRII	184.05 ± 156.71	105.70	168.38	246.74	141.04
Soluble vascular endothelial growth factor receptor 1	sVEGFR-1	255.90 ± 141.30	185.25	241.77	312.42	127.17
Soluble vascular endothelial growth factor receptor 2	sVEGFR-2	106.68 ± 110.78	51.29	95.60	150.99	99.70
Soluble vascular endothelial growth factor receptor 3	sVEGFR-3	231.15 ± 128.39	166.96	218.31	282.50	115.55
Soluble hepatocyte growth factor receptor/cMET	sHGFR/cMET	298.17 ± 295.54	150.40	268.62	416.39	265.99
Soluble AXL	sAXL	49.01 ± 34.92	31.55	45.52	62.97	31.42
Osteopontin	OPN	80.36 ± 79.68	40.52	72.39	112.24	71.71
Soluble platelet endothelial cell adhesion molecule 1	sPECAM-1	27.46 ± 28.82	13.05	24.58	38.99	25.94
Soluble HER2	sHER-2	282.10 ± 215.68	174.26	260.53	368.38	194.11
Soluble HER3	sHER-3	270.88 ± 149.18	196.29	255.96	330.55	134.26
Soluble urokinase plasminogen activator receptor	suPAR	228.82 ± 103.69	176.98	218.45	270.30	93.32
Soluble Tie2	sTie-2	181.53 ± 151.80	105.62	166.35	242.25	136.62
Soluble interleukin-6 receptor alpha	sIL-6Rα	307.56 ± 182.02	216.56	289.36	380.37	163.81
Soluble neuropilin-1	sNRP-1	309.95 ± 311.90	154.00	278.76	434.71	280.71
Soluble E-selectin	sE-selectin	388.96 ± 117.83	330.05	377.18	436.09	106.04
Platelet-derived growth factor-AB/BB	PDGF-AB/BB	252.80 ± 114.65	195.48	241.34	298.66	103.18
Thrombospondin-2	THBS-2	410.53 ± 338.43	241.31	376.69	545.90	304.59

Supplementary Table S4. Biomarker measurements at Day 112 — plasma concentrations (pg/ml) for all 88 circulating cytokines.

**Supplementary Table S4. Biomarker measurements at Day 112. Complete plasma concentrations (pg/ml) for all 88 circulating cytokines. Values are mean ± standard deviation (SD) and percentiles. n = 15 (PD: 3, SD: 12). IQR: interquartile range (25th–75th percentile).**						
Analyzed circulating protein	Acronym	Mean (pg/ml) ± SD	P25	Median	P75	IQR
Epidermal growth factor	EGF	26.07 ± 21.66	15.24	23.90	34.73	19.49
Fibroblast growth factor-2	FGF-2	14.56 ± 11.10	9.01	13.45	19.00	9.99
Eotaxin-1	Eotaxin-1	488.44 ± 274.12	351.38	461.03	598.09	246.71
Transforming growth factor alpha	TGFα	487.24 ± 425.08	274.70	444.73	657.27	382.57
Granulocyte colony-stimulating factor	G-CSF	442.57 ± 140.61	372.27	428.51	498.82	126.55
Fms-like tyrosine kinase 3 ligand	FLT-3L	369.14 ± 282.41	227.94	340.90	482.11	254.17
Granulocyte-Macrophage Colony-Stimulating Factor	GM-CSF	304.15 ± 194.71	206.80	284.68	382.04	175.24
Fractalkine	Fractalkine	488.60 ± 434.73	271.24	445.13	662.50	391.26
Interferon alpha-2	IFNα2	364.16 ± 333.18	197.57	330.84	497.43	299.86
Interferon-gamma	IFNγ	275.45 ± 284.28	133.31	247.02	389.16	255.86
Growth-Regulated Oncogene	GRO	310.05 ± 250.75	184.68	284.98	410.35	225.68
Interleukin-10	IL-10	74.01 ± 59.26	44.38	68.09	97.72	53.34
Monocyte Chemotactic Protein-3	MCP-3	81.45 ± 67.42	47.75	74.71	108.42	60.67
Interleukin-12P40	IL-12P40	216.10 ± 213.12	109.54	194.79	301.35	191.81
Macrophage-Derived Chemokine	MDC	296.79 ± 186.55	203.52	278.13	371.41	167.89
Interleukin-12P70	IL-12P70	261.48 ± 130.15	196.40	248.46	313.54	117.14
Interleukin-13	IL-13	490.91 ± 525.20	228.31	438.39	700.99	472.68
Interleukin-15	IL-15	200.68 ± 99.52	150.93	190.73	240.49	89.56
Soluble CD40L	sCD40L	225.87 ± 197.52	127.11	206.12	304.88	177.76
Interleukin-17a	IL-17a	225.63 ± 258.82	96.22	199.74	329.15	232.94
Interleukin-1 receptor antagonist	IL-1Ra	30.11 ± 18.28	20.97	28.28	37.42	16.46
Interleukin-1alpha	IL-1α	260.74 ± 88.92	216.28	251.85	296.31	80.03
Interleukin-9	IL-9	235.81 ± 192.22	139.70	216.59	312.70	173.00
Interleukin-1beta	IL-1β	155.42 ± 185.65	62.60	136.86	229.69	167.09
Interleukin-2	IL-2	177.88 ± 112.25	121.76	166.66	222.78	101.03

Interleukin-3	IL-3	299.43 ± 161.14	218.86	283.32	363.89	145.02
Interleukin-4	IL-4	238.23 ± 105.00	185.72	227.72	280.23	94.50
Interleukin-5	IL-5	32.67 ± 29.94	17.70	29.68	44.65	26.94
Interleukin-6	IL-6	142.78 ± 63.51	111.03	136.43	168.18	57.16
Interleukin-7	IL-7	132.70 ± 89.53	87.93	123.75	168.51	80.58
Interleukin-8	IL-8	77.69 ± 71.90	41.74	70.50	106.45	64.71
Interferon gamma-induced protein-10	IP-10	362.49 ± 425.26	149.86	319.96	532.59	382.73
Monocyte chemoattractant protein-1	MCP-1	26.11 ± 15.24	18.49	24.58	32.20	13.71
Macrophage inflammatory protein-1 alpha	MIP-1α	338.10 ± 358.65	158.78	302.24	481.56	322.78
Macrophage Inflammatory Protein-1 beta	MIP-1β	127.48 ± 137.69	58.63	113.71	182.56	123.92
Tumor necrosis factor alpha	TNFα	25.79 ± 20.44	15.57	23.75	33.97	18.40
Tumor necrosis factor beta	TNFβ	16.92 ± 7.50	13.18	16.17	19.92	6.75
Vascular endothelial growth factor	VEGF	358.92 ± 252.00	232.92	333.72	459.73	226.80
Eotaxin-2	Eotaxin-2	455.70 ± 521.70	194.85	403.53	664.38	469.53
Monocyte chemoattractant protein-2	MCP-2	226.00 ± 176.20	137.90	208.38	296.48	158.58
B-cell-attracting chemokine-1	BCA-1	192.33 ± 86.51	149.07	183.67	226.93	77.86
Monocyte chemoattractant protein-4	MCP-4	212.16 ± 215.82	104.25	190.58	298.49	194.24
I-309	I-309	208.85 ± 213.81	101.94	187.47	294.37	192.43
Interleukin-16	IL-16	71.95 ± 28.58	57.66	69.09	83.38	25.72
Thymus and Activation-Regulated Chemokine	TARC	490.25 ± 249.85	365.33	465.27	590.19	224.86
6CKINE	6CKINE	75.72 ± 83.10	34.18	67.41	108.96	74.79
Eotaxin-3	Eotaxin-3	378.78 ± 414.05	171.76	337.38	544.40	372.65
Leukemia inhibitory factor	LIF	432.86 ± 177.14	344.29	415.15	503.71	159.43
Thrombopoietin	TPO	289.37 ± 254.29	162.22	263.94	391.08	228.86
Stem cell factor	SCF	136.17 ± 114.96	78.69	124.67	182.15	103.46
Thymic stromal lymphopoietin	TSLP	180.14 ± 105.85	127.22	169.56	222.48	95.27
Interleukin-33	IL-33	200.65 ± 160.28	120.51	184.62	264.76	144.25
Interleukin-20	IL-20	95.96 ± 92.49	49.72	86.71	132.96	83.24
Interleukin-21	IL-21	187.65 ± 123.44	125.93	175.31	237.02	111.09
Interleukin-23	IL-23	211.09 ± 170.86	125.66	194.01	279.44	153.77
TNF-related apoptosis-inducing ligand	TRAIL	482.06 ± 529.09	217.52	429.15	693.69	476.18
Cutaneous T cell-attracting chemokine	CTACK	261.79 ± 107.31	208.14	251.06	304.72	96.58
Stromal cell-derived factor-1 alpha+beta	SDF-1α+β	41.86 ± 44.52	19.61	37.41	59.67	40.06
Epithelial-derived neutrophil-activating peptide-78	ENA-78	9.70 ± 5.27	7.07	9.18	11.81	4.74
Macrophage inflammatory protein-1 delta	MIP-1D	256.93 ± 78.46	217.70	249.09	288.31	70.61
Interleukin-28a	IL-28a	321.44 ± 353.56	144.66	286.09	462.87	318.20
Soluble CD30	sCD30	333.36 ± 310.69	178.02	302.30	457.64	279.62
Soluble epidermal growth factor receptor	sEGFR	365.67 ± 317.82	206.76	333.89	492.80	286.04
Soluble glycoprotein 130	sgp130	10.61 ± 3.51	8.86	10.26	12.02	3.16
Soluble	sIL-1RI	380.65 ± 374.56	193.36	343.19	530.47	337.11

interleukin-1 receptor I						
Soluble interleukin-1 receptor II	sIL-1RII	414.63 ± 397.65	215.81	374.87	573.69	357.88
Soluble interleukin-2 receptor alpha	sIL-2Rα	122.23 ± 39.09	102.68	118.32	137.86	35.18
Soluble interleukin-4 receptor	sIL-4R	277.66 ± 93.89	230.71	268.27	315.22	84.51
Soluble interleukin-6 receptor	sIL-6R	333.65 ± 192.98	237.16	314.35	410.84	173.68
Soluble receptor for advanced glycation endproducts	sRAGE	347.11 ± 338.21	178.01	313.29	482.40	304.39
Soluble tumor necrosis factor receptor type I	sTNFRI	471.87 ± 361.40	291.18	435.73	616.43	325.26
Soluble tumor necrosis factor receptor type II	sTNFRII	241.95 ± 111.65	186.12	230.78	286.60	100.48
Soluble vascular endothelial growth factor receptor 1	sVEGFR-1	129.98 ± 44.48	107.75	125.54	147.77	40.03
Soluble vascular endothelial growth factor receptor 2	sVEGFR-2	483.25 ± 209.94	378.28	462.26	567.23	188.94
Soluble vascular endothelial growth factor receptor 3	sVEGFR-3	378.06 ± 120.00	318.06	366.06	426.06	108.00
Soluble hepatocyte growth factor receptor/cMET	sHGFR/cMET	233.05 ± 108.98	178.57	222.16	276.64	98.08
Soluble AXL	sAXL	379.53 ± 155.45	301.81	363.99	441.71	139.90
Osteopontin	OPN	381.65 ± 389.88	186.71	342.66	537.60	350.89
Soluble platelet endothelial cell adhesion molecule 1	sPECAM-1	275.76 ± 216.34	167.59	254.13	362.30	194.71
Soluble HER2	sHER-2	224.43 ± 208.06	120.40	203.62	307.65	187.25
Soluble HER3	sHER-3	225.89 ± 245.20	103.29	201.37	323.97	220.68
Soluble urokinase plasminogen activator receptor	suPAR	94.90 ± 38.55	75.63	91.04	110.32	34.69
Soluble Tie2	sTie-2	297.15 ± 139.95	227.17	283.15	353.13	125.96
Soluble interleukin-6 receptor alpha	sIL-6Rα	101.53 ± 87.91	57.57	92.74	136.69	79.12
Soluble neuropilin-1	sNRP-1	489.22 ± 207.53	385.45	468.46	572.23	186.78
Soluble E-selectin	sE-selectin	136.05 ± 71.10	100.50	128.94	164.49	63.99
Platelet-derived growth factor-AB/BB	PDGF-AB/BB	175.73 ± 153.83	98.81	160.34	237.26	138.45
Thrombospondin-2	THBS-2	274.33 ± 137.31	205.68	260.60	329.26	123.58

Supplementary Table S5. Baseline (Day 0) plasma concentrations (pg/ml) for all 88 circulating cytokines.

**Supplementary Table S5. Biomarker measurements at Day 0 (baseline). Complete plasma concentrations (pg/ml) for all 88 circulating cytokines. Values are mean ± standard deviation (SD) and percentiles. n = 34. IQR: interquartile range (25th–75th percentile).**									
Analyzed circulating protein	Acronym	Other acronyms	Mean (pg/ml) ± SD	P25	Median	P75	IQR		
Epidermal growth factor	EGF		113.90 ± 264.46	29.52	41.55	72.55	43.03		
Fibroblast growth factor-2	FGF-2	bFGF	63.45 ± 8.85	59.40	60.80	65.05	5.65		
Eotaxin-1	Eotaxin-1	CCL-11	583.02 ± 701.81	281.95	422.14	594.45	312.50		
Transforming growth factor alpha	TGFα		9.86 ± 9.44	3.21	7.60	14.01	10.81		
Granulocyte colony-stimulating factor	G-CSF	CSF-3	28.97 ± 23.48	18.53	23.23	27.94	9.40		
Fms-like tyrosine kinase 3 ligand	FLT-3L	FLT3LG	25.98 ± 37.27	2.09	8.27	38.53	36.44		
Granulocyte-Macrophage Colony-Stimulating Factor	GM-CSF	CSF-2	18.55 ± 14.01	10.65	15.07	19.79	9.15		
Fractalkine	Fractalkine	CX3CL1	245.16 ± 994.82	39.17	57.65	99.21	60.04		
Interferon alpha-2	IFNα2		50.37 ± 15.50	40.23	45.15	52.97	12.74		
Interferon-gamma	IFNγ		10.13 ± 13.29	4.26	6.54	9.90	5.64		
Growth-Regulated Oncogene	GRO		3443.66 ± 1756.35	1981.16	2723.24	4598.68	2617.52		
Interleukin-10	IL-10		6.75 ± 18.75	0.00	0.00	1.83	1.83		
Monocyte Chemotactic Protein-3	MCP-3	CCL-7	44.96 ± 71.85	23.85	24.82	26.55	2.70		
Interleukin-12P40	IL-12P40		13.40 ± 58.07	0.00	0.00	4.05	4.05		
Macrophage-Derived Chemokine	MDC	CCL-22	375.05 ± 224.42	178.56	337.02	551.89	373.33		
Interleukin-12P70	IL-12P70		1.73 ± 3.86	0.00	0.00	1.64	1.64		
Interleukin-13	IL-13		16.88 ± 67.24	0.00	0.00	0.00	0.00		
Interleukin-15	IL-15		6.32 ± 7.91	3.48	5.01	6.09	2.62		
Soluble CD40L	sCD40L		1182.63 ± 1696.64	448.45	682.06	1144.04	695.59		
Interleukin-17a	IL-17a		5.74 ± 3.77	3.94	4.87	5.68	1.75		
Interleukin-1	IL-1Ra	IL-1RN	45.26 ±	0.00	0.00	0.00	0.00		

receptor antagonist			172.28						
Interleukin-1alpha	IL-1α		29.00 ± 38.73	6.95	18.28	31.80	24.85		
Interleukin-9	IL-9		0.84 ± 4.90	0.00	0.00	0.00	0.00		
Interleukin-1beta	IL-1β		3.44 ± 1.39	2.83	3.06	3.58	0.76		
Interleukin-2	IL-2		2.11 ± 5.91	0.63	1.07	1.36	0.73		
Interleukin-3	IL-3		5072.64 ± 6.82	5072.81	5074.16	5074.96	2.15		
Interleukin-4	IL-4		0.87 ± 4.94	0.00	0.00	0.00	0.00		
Interleukin-5	IL-5		2.50 ± 7.82	0.00	0.00	0.00	0.00		
Interleukin-6	IL-6		15.55 ± 10.10	10.32	12.63	17.44	7.12		
Interleukin-7	IL-7		22.08 ± 0.89	21.55	21.73	22.36	0.81		
Interleukin-8	IL-8		2321.01 ± 3161.59	41.62	111.69	5718.03	5676.41		
Interferon gamma-induced protein-10	IP-10	CXCL-10	860.20 ± 355.13	637.41	860.67	1017.86	380.45		
Monocyte chemoattractant protein-1	MCP-1	CCL-2	837.05 ± 375.51	558.52	820.91	1037.08	478.56		
Macrophage inflammatory protein-1 alpha	MIP-1α	CCL-3	5237.02 ± 450.11	5231.35	5455.83	5465.56	234.21		
Macrophage Inflammatory Protein-1 beta	MIP-1β	CCL-4	98.84 ± 154.98	40.55	45.61	87.13	46.58		
Tumor necrosis factor alpha	TNFα		79.23 ± 141.10	12.69	37.27	90.48	77.79		
Tumor necrosis factor beta	TNFβ	LT-α	41.78 ± 139.08	0.00	0.00	0.00	0.00		
Vascular endothelial growth factor	VEGF	VPF	83.93 ± 33.29	68.07	73.62	83.81	15.73		
Eotaxin-2	Eotaxin-2	CCL-24	517.82 ± 329.27	223.22	517.58	708.26	485.04		
Monocyte chemoattractant protein-2	MCP-2	CCL-8	25.57 ± 2.54	24.00	24.59	26.16	2.16		
B-cell-attracting chemokine-1	BCA-1	CXCL-13	73.33 ± 169.96	21.17	30.27	50.49	29.33		
Monocyte chemoattractant protein-4	MCP-4	CCL-13	73.74 ± 41.99	45.31	56.08	89.14	43.82		
I-309	I-309	CCL-1	7.33 ± 0.35	7.16	7.27	7.38	0.22		
Interleukin-16	IL-16		137.79 ± 159.28	91.82	95.07	102.56	10.74		
Thymus and Activation-Regulated Chemokine	TARC	CCL-17	47.78 ± 52.30	16.20	27.90	48.88	32.69		
6CKINE	6CKINE	CCL-21, SLC	210.98 ± 70.17	172.42	192.34	226.70	54.29		
Eotaxin-3	Eotaxin-3	CCL-26	107.29 ± 66.91	86.52	94.03	101.99	15.48		
Leukemia inhibitory factor	LIF	CDF	4.88 ± 4.38	2.82	3.59	5.70	2.88		
Thrombopoietin	TPO	THPO, MGDF	517.09 ± 1000.06	219.29	316.89	448.10	228.80		
Stem cell factor	SCF	KITLG, KL-1	32.90 ± 22.80	21.54	26.78	38.38	16.84		
Thymic stromal lymphopoietin	TSLP		179.88 ± 1035.22	0.00	0.00	0.00	0.00		
Interleukin-33	IL-33		12.46 ± 43.90	0.48	2.77	5.34	4.86		
Interleukin-20	IL-20		260.67 ± 19.81	252.10	255.26	259.49	7.39		
Interleukin-21	IL-21		0.94 ±	0.00	0.00	0.00	0.00		

			3.57							
Interleukin-23	IL-23		221.80 ± 725.15	0.00	52.66	118.15	118.15		
TNF-related apoptosis-inducing ligand	TRAIL	TNFSF-10	49.41 ± 31.52	34.56	44.92	63.45	28.89		
Cutaneous T cell-attracting chemokine	CTACK		1153.68 ± 379.68	934.60	1154.82	1304.93	370.33		
Stromal cell-derived factor-1 alpha+beta	SDF-1α+β	CXCL-12	1924.68 ± 675.83	1361.72	1707.42	2573.68	1211.96		
Epithelial-derived neutrophil-activating peptide-78	ENA-78	CXCL-5	552.76 ± 689.00	178.00	260.71	513.57	335.57		
Macrophage inflammatory protein-1 delta	MIP-1D	CCL-15	4686.69 ± 2379.29	3357.19	4700.68	5440.99	2083.80		
Interleukin-28a	IL-28a		97.18 ± 313.03	10.28	16.55	35.34	25.06		
Soluble CD30	sCD30		67.43 ± 9.44	61.09	67.62	74.15	13.06		
Soluble epidermal growth factor receptor	sEGFR		24334.40 ± 9315.22	18957.05	23139.30	28322.60	9365.55		
Soluble glycoprotein 130	sgp130		44580.11 ± 9980.83	37428.05	44151.70	52851.60	15423.55		
Soluble interleukin-1 receptor I	sIL-1RI	sCD121α	61.04 ± 7.57	57.16	60.08	64.75	7.59		
Soluble interleukin-1 receptor II	sIL-1RII		1304.63 ± 926.28	723.64	1090.63	1486.98	763.33		
Soluble interleukin-2 receptor alpha	sIL-2Rα	sCD25	257.57 ± 120.89	174.18	237.76	278.94	104.76		
Soluble interleukin-4 receptor	sIL-4R		245.62 ± 8.14	240.41	244.34	248.56	8.15		
Soluble interleukin-6 receptor	sIL-6R		6560.42 ± 2361.00	4939.43	6403.40	7918.17	2978.74		
Soluble receptor for advanced glycation endproducts	sRAGE		74.29 ± 37.36	57.67	71.36	75.93	18.26		
Soluble tumor necrosis factor receptor type I	sTNFRI		258.07 ± 182.50	150.17	201.49	282.47	132.30		
Soluble tumor necrosis factor receptor type II	sTNFRII		2184.49 ± 1149.96	1378.54	1811.82	2746.49	1367.95		
Soluble vascular endothelial growth factor receptor 1	sVEGFR-1		71122.51 ± 140802.72	472.49	516.84	576.02	103.53		
Soluble vascular endothelial growth factor receptor 2	sVEGFR-2		1760.73 ± 636.97	1361.15	1658.82	2101.95	740.80		
Soluble vascular endothelial growth factor receptor 3	sVEGFR-3		338.00 ± 107.60	262.34	339.94	384.29	121.95		
Soluble hepatocyte growth factor receptor/cMET	sHGFR/cMET		6807.43 ± 1489.53	5753.41	6838.36	7603.51	1850.10		
Soluble AXL	sAXL		588.95 ± 260.73	408.06	499.08	766.30	358.24		
Osteopontin	OPN		354.57 ± 278.07	152.80	342.54	511.54	358.74		
Soluble platelet endothelial cell adhesion molecule 1	sPECAM-1		166.62 ± 44.54	138.27	165.48	192.08	53.81		

Soluble HER2	sHER-2		185.20 ± 56.64	142.26	186.93	219.84	77.58		
Soluble HER3	sHER-3		179.39 ± 108.46	90.66	160.54	228.81	138.16		
Soluble urokinase plasminogen activator receptor	suPAR		91.22 ± 59.12	52.66	73.26	127.94	75.28		
Soluble Tie2	sTie-2		390.80 ± 233.51	216.40	327.69	545.85	329.46		
Soluble interleukin-6 receptor alpha	sIL-6Rα		1049.87 ± 257.05	906.49	1037.60	1221.67	315.18		
Soluble neuropilin-1	sNRP-1		1189.92 ± 181.46	1051.36	1189.70	1317.76	266.40		
Soluble E-selectin	sE-selectin		180.52 ± 127.95	90.93	138.37	224.04	133.10		
Platelet-derived growth factor-AB/BB	PDGF-AB/BB		272.10 ± 471.92	49.93	108.76	253.10	203.17		
Thrombospondin-2	THBS-2		245.45 ± 269.39	85.06	181.85	269.29	184.23		

Supplementary Table S6. Exploratory subgroup analysis: CRC patients only (n=30). Univariate Cox proportional hazards regression for PFS.

**Supplementary Table S6. Exploratory subgroup analysis: CRC patients only (n=30).**						
Univariate Cox proportional hazards regression for progression-free survival (PFS) in the colorectal cancer subgroup. Results are consistent with the full-cohort analysis (Table 5). ECOG PS: Eastern Cooperative Oncology Group Performance Status. HR: Hazard Ratio. CI: Confidence Interval. * p < 0.05.						
A. Patient characteristics — CRC subgroup (n=30)						
Characteristic	n	%				
Total patients	30	100%				
Progressive disease (PD)	18	60.0%				
Stable disease (SD)	12	40.0%				
ECOG PS 0	11	36.7%				
ECOG PS 1	18	60.0%				
ECOG PS 2	1	3.3%				
B. Univariate Cox regression — PFS (CRC subgroup, n=30)						
Variable	Comparison	n events/total	HR	95% CI	p-value	Full cohort p
ECOG PS	≥1 vs 0	17/30	4.74	1.35–16.65	0.015*	0.021*
Tumor type	CRC vs all (reference)	—	—	—	—	0.367
C. Survival by ECOG PS — CRC subgroup						
Group	n	Median PFS (months)		Median OS (months)		Log-rank p (PFS)
ECOG PS 0	11	4.0		12.8		
ECOG PS 1–2	19	2.0		5.2		0.015*
Note: These analyses are exploratory and limited by the reduced sample size (n=30). The non-CRC subgroup (n=8: 3 biliary tract, 2 hepatocellular, 2						

pancreatic, 1 gastric) was too small for independent statistical analysis. Tumor type (CRC vs non-CRC) was not a significant prognostic factor in either univariate (HR=1.30, p=0.634) or multivariate (HR=1.66, p=0.367) analysis of the full cohort, supporting the retention of the mixed-histology cohort in the primary analysis. Kaplan-Meier curves for this subgroup are available upon request.						

Supplementary Table S7. Assay sensitivities and precision of investigated circulating biomarkers (Luminex® multiplex immunoassay).

MinDC: minimum detectable concentration (pg/mL). Intra-assay %CV: mean of %CVs from eight reportable results across two concentrations in a single assay. Inter-assay %CV: mean of %CVs across two concentrations over six experiments (N=6). Key biomarkers used in the prognostic panels are highlighted.

**Supplementary Table S7. Assay sensitivities and precision of investigated circulating biomarkers (Luminex® multiplex immunoassay platform, MerckMillipore HCYTOMAG-60K, HCYP2MAG-62K, HSCRMAG-32K and HANG2MAG-12K kits).**					
MinDC: minimum detectable concentration (pg/mL). Intra-assay %CV: mean of %CV from eight reportable results across two concentrations in a single assay. Inter-assay %CV: mean of %CV across two concentrations over six experiments (N=6). Key biomarkers used in the prognostic panels are highlighted in bold.					
Cytokine	MinDC (pg/mL)	MinDC+2SD (pg/mL)	Intra-assay %CV	Inter-assay %CV (N=6 assays)	Key biomarker
EGF	2.8	4.6	2.3	5.8	
FGF-2	7.6	11.8	2.3	4.8	
Eotaxin	4.0	6.8	7.2	10.8	★
TGFα	0.8	1.2	4.1	9.5	
G-CSF	1.8	3.3	1.8	15.5	
Flt-3L	5.4	7.0	2.4	6.6	
GM-CSF	7.5	15.0	3.1	10.1	
Fractalkine	22.7	37.7	4.5	9.4	
IFNα2	2.9	4.8	2.4	13.3	
IFNγ	0.8	1.1	1.6	12.0	
GRO	9.9	14.1	2.1	9.2	
IL-10	1.1	1.6	1.6	16.8	
MCP-3	3.8	6.4	1.6	6.4	
IL-12P40	7.4	12.7	2.8	12.4	
MDC	3.6	7.1	1.6	7.2	
IL-12P70	0.6	1.0	2.2	16.7	
IL-13	1.3	1.9	2.2	9.2	
IL-15	1.2	1.7	2.7	8.1	

sCD40L	5.1	9.9	3.7	18.9	
IL-17A	0.7	1.2	2.2	7.9	
IL-1RA	8.3	17.1	2.1	10.7	
IL-1α	9.4	12.6	3.3	12.8	
IL-9	1.2	2.0	2.4	8.4	
IL-1β	0.8	1.0	2.3	6.7	
IL-2	1.0	1.6	2.1	6.3	
IL-3	0.7	1.0	3.4	6.1	
IL-4	4.5	7.1	2.9	14.2	
IL-5	0.5	0.7	2.6	10.8	
IL-6	0.9	1.3	2.0	18.3	
IL-7	1.4	2.4	1.7	16.1	
IL-8	0.4	0.7	1.9	3.5	
IP-10	8.6	14.0	2.6	15.3	
MCP-1	1.9	3.4	1.5	7.9	
MIP-1α	2.9	6.2	1.9	14.5	
MIP-1β	3.0	4.8	2.4	8.8	
TNFα	0.7	1.1	2.6	13.0	
TNFβ	1.5	1.9	1.6	11.4	
VEGF	26.3	47.9	3.7	10.4	
sAXL	2.7	5.6	<10%	<15%	
sHer2	5.0	11.9	<10%	<15%	
sHer3	6.1	17.9	<10%	<15%	
sE-Selectin	116.6	247.8	<10%	<15%	
sHGFR/c-Met	10.4	24.2	<10%	<15%	
PDGF-AB/BB	6.4	24.2	<10%	<15%	★
sIL-6Rα	7.4	15.1	<10%	<15%	
sTie-2	12.2	35.9	<10%	<15%	
Thrombospondin-2	23.5	118.6	<10%	<15%	★
sNeuropilin-1	54.9	151.0	<10%	<15%	
suPAR	36.9	129.5	<10%	<15%	
sPECAM-1	6.3	15.5	<10%	<15%	
Osteopontin	29.6	73.9	<10%	<15%	
sCD30	7.0	14.7	<10%	<15%	
sEGFR	42.0	60.7	<10%	<15%	
sgp130	6.0	6.9	<10%	<15%	
sIL-1RI	21.0	22.0	<10%	<15%	
sIL-1RII	115.0	115.6	<10%	<15%	
sIL-2Rα	11.0	20.9	<10%	<15%	
sIL-4R	14.0	17.9	<10%	<15%	
sIL-6R	9.0	14.1	<10%	<15%	
sRAGE	3.0	3.8	<10%	<15%	
sTNFRI	12.0	14.3	<10%	<15%	
sTNFRII	8.0	19.2	<10%	<15%	
sVEGFR1	111.0	208.4	<10%	<15%	
sVEGFR2	71.0	116.4	<10%	<15%	
sVEGFR3	47.0	121.9	<10%	<15%	
Eotaxin-2	—	4.4	4.5	8.1	★
MCP-2	—	2.2	4.5	7.3	

BCA-1	—	1.3	4.8	6.2	
MCP-4	—	3.4	3.6	6.1	★
I-309	—	1.4	7.0	11.0	
IL-16	—	9.1	3.5	16.6	★
TARC	—	0.4	5.6	9.8	
6CKine	—	45.4	8.6	13.7	
Eotaxin-3	—	8.7	8.3	14.5	★
LIF	—	5.8	6.0	12.1	
TPO	—	37.9	8.6	9.8	
SCF	—	5.6	7.4	11.6	
TSLP	—	3.1	6.9	13.8	
IL-33	—	6.2	5.2	7.2	
IL-20	—	53.3	4.6	7.6	
IL-21	—	6.8	5.6	12.7	
IL-23	—	31.5	4.6	7.5	
TRAIL	—	3.5	5.3	10.2	★
CTACK	—	1.8	8.2	9.2	
SDF-1α+β	—	55.8	11.1	9.2	
ENA-78	—	7.2	4.3	6.2	
MIP-1δ	—	10.5	5.3	12.0	
IL-28A	—	7.9	4.4	10.1	

Supplementary Table S8. Accuracy and spike recovery of the evaluated cytokines.

Values represent mean percent recovery of spiked standards at low, medium, and high concentrations in plasma matrices (n=5). Acceptable range: 70–130%.

**Supplementary Table S8. Accuracy and spike recovery of the evaluated cytokines (MerckMillipore HCYTOMAG-60K, HCYP2MAG-62K, HSCRMAG-32K and HANG2MAG-12K kits).**	
Values represent mean percent recovery of spiked standards at low, medium, and high concentrations in plasma matrices (n=5). Acceptable range: 70–130%. Key biomarkers used in the prognostic panels are indicated with ★.	
Cytokine	% Recovery in plasma matrix
EGF	97.5
FGF-2	99.0
★ Eotaxin	100.5
TGFα	91.7
G-CSF	100.3
Flt-3L	98.2
GM-CSF	100.7
Fractalkine	87.2
IFNα2	93.9
IFNγ	98.1
GRO	97.5
IL-10	97.7
MCP-3	97.0
IL-12P40	93.3
MDC	102.3
IL-12P70	104.0
IL-13	95.0
IL-15	95.3
sCD40L	95.2
IL-17A	103.8
IL-1RA	93.5
IL-1α	92.9
IL-9	99.4
IL-1β	94.9
IL-2	95.4
IL-3	101.0
IL-4	94.5
IL-5	99.9
IL-6	96.1
IL-7	93.0
IL-8	98.3
IP-10	93.8
MCP-1	98.3
MIP-1α	105.0
MIP-1β	92.4
TNFα	97.8
TNFβ	97.5
VEGF	91.8
sAXL	96
sHer2	94
sHer3	88
sE-Selectin	88
sHGFR/c-Met	96
★ PDGF-AB/BB	95
sIL-6Rα	96
sTie-2	96
★ Thrombospondin-2	95
sNeuropilin-1	95
suPAR	92
sPECAM-1	96
Osteopontin	97
sCD30	101
sEGFR	101
sgp130	103
sIL-1RI	103
sIL-1RII	96
sIL-2Rα	91
sIL-4R	104
sIL-6R	103

sRAGE	104
sTNFRI	97
sTNFRII	99
sVEGFR1	97
sVEGFR2	101
sVEGFR3	96
★ Eotaxin-2	100.4
MCP-2	106.2
BCA-1	101.3
★ MCP-4	104.6
I-309	103.4
★ IL-16	96.6
TARC	100.9
6CKine	93.3
★ Eotaxin-3	104.2
LIF	102.0
TPO	99.7
SCF	99.4
TSLP	109.5
IL-33	103.9
IL-20	98.5
IL-21	99.8
IL-23	99.7
★ TRAIL	102.9
CTACK	98.5
SDF-1α+β	81.3
ENA-78	107.9
MIP-1δ	94.1
IL-28A	Not reported

Supplementary Figure S1. Kaplan-Meier survival curves for subgroup analyses.

(A) PFS by ECOG PS — full cohort (n=38). (B) OS by ECOG PS — full cohort. (C) PFS by ECOG PS — CRC subgroup (n=30). (D) PFS: CRC vs non-CRC.

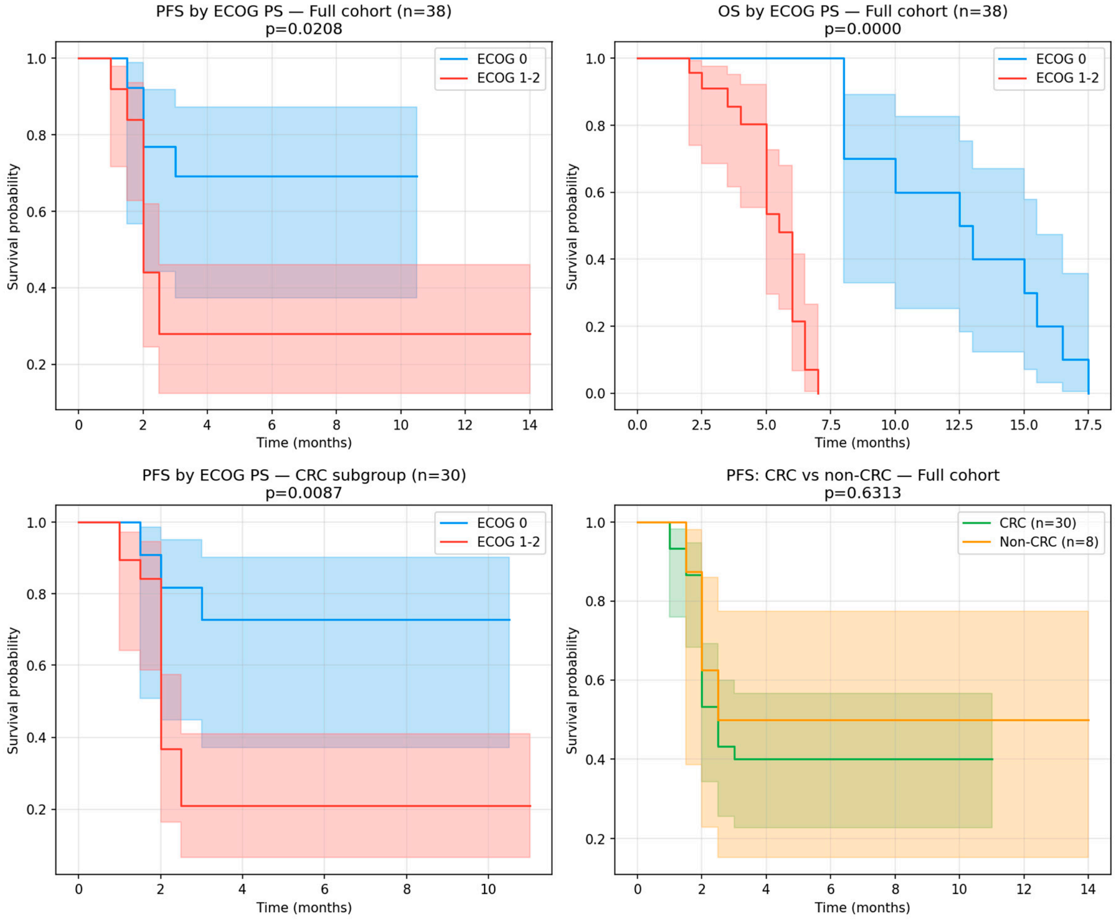

Supplement: Supplementary file 1 [file cancers-18-01762-s001.zip › cancers-4290816-supplementary.pdf]
